# Immunotherapy-Mediated Modulation of the Gut Microbiota in Multiple Sclerosis: The Effects of High-Efficacy (Cladribine) and Moderate-Efficacy (Interferon Beta-1a) Treatments

**DOI:** 10.3390/ijms27083500

**Published:** 2026-04-14

**Authors:** Elsebeth Staun-Ram, Anat Volkowich, Lea Glass-Marmor, Ariel Miller

**Affiliations:** 1 Rappaport Faculty of Medicine, Technion-Israel Institute of Technology, Haifa 3109601, Israel; elsebeth@technion.ac.il; 2 Neuroimmunology Unit & Multiple Sclerosis Center, Lady Davis Carmel Medical Center, Haifa 3436212, Israel; 3 Department of Neurology, Lady Davis Carmel Medical Center, Haifa 3436212, Israel

**Keywords:** adverse events, cladribine, clinical response, disease modifying therapy, efficacy, interferon-β, microbiota, multiple sclerosis, SCFAs

## Abstract

Interactions between the gut microbiota, immune system, and brain seem to be involved in the pathogenesis and disease activity of multiple sclerosis (MS). Some MS disease-modifying therapies (DMTs) have been shown to alter the microbiota, but whether this is related to their specific mode of action or indirectly related to their immune-modulatory effect is unknown. In this longitudinal study, we characterized the effects of two DMTs on the microbiota under similar conditions and populations: the injectable, moderate-efficacy DMT interferon beta-1a (INFβ-1a) and the oral, high-efficacy DMT cladribine tablets (CladT). Taxonomic differences were identified following 6 months of therapy for each DMT, and both were associated with the elevation of short-chain fatty acid (SCFA) producers from the *Lachnospiraceae*, *Lactobacillaceae*, and *Ruminococcaceae* families (Firmicutes), while members of Bacteroidetes and Proteobacteria were reduced. Moreover, a higher abundance of Alphaproteobacteria and Betaproteobacteria at baseline was associated with disease activity within 1–2 years of follow-up, while a higher abundance of *Lachnospiraceae*, *Ruminococcaceae*, *Bifidobacteriaceae*, and *Streptococcaceae* microbes, among others, was associated with no evidence of disease activity (NEDA). Our results provide supporting evidence that alteration of the microbiota by DMTs is part of their beneficial effect, and while some modifications seem to be DMT-specific, MS-DMTs in general promote SCFA-producing microbes, which positively correlate with a favorable clinical outcome. Future therapeutic strategies for PwMS may benefit from microbiome modulation, contingent upon additional mechanistic and interventional studies.

## 1. Introduction

Ongoing efforts to elucidate the underlying mechanisms of multiple sclerosis (MS) pathogenesis and disease progression (as a chronic, neurodegenerative autoimmune disease with a multi-factorial background) have led, among other things, to the study of the role of the gut microbiome in MS. The gut microbiota plays an important role in maintaining healthy processes, such as food metabolism, energy homeostasis, vitamin production, intestinal barrier integrity, and the development and activity of the immune system [1]. The commercial gut microbiota produces various metabolites that interact with the host, such as short-chain fatty acids (SCFAs) from the fermentation of dietary fibers, mainly acetate, propionate, and butyrate, which have well-known anti-inflammatory effects, and metabolites from the metabolism of tryptophan, phytoestrogens, choline, and bile acids [1,2,3]. The composition of an individual’s microbiota is shaped during early childhood and influenced by both genetics and early-life factors, and it remains relatively stable throughout life, although it may be temporarily or permanently affected by environmental factors such as culture, diet, geographic exposure, lifestyle, infections, medications, etc. [4]. Dysregulated composition of the microbiota, known as dysbiosis, with reduced SCFA-producing bacteria, altered energy and gut hormone regulation, increased intestinal permeability, translocation of bacterial metabolites, and systemic immune responses, has, together with molecular mimicry, been suggested to play a role in the development of autoimmune diseases including MS [1,5,6]. Bidirectional interactions between the gastrointestinal tract and the central nervous system (CNS)—the brain−gut axis—occur through several routes, including the sympathetic and parasympathetic nervous systems and the neuroendocrine and immune systems, as well as through microbial-produced signaling molecules [7,8]. Numerous reports, including from our group, have found that while the general diversity (α- and β-diversity) may not differ between patients with MS (PwMS) and healthy individuals, the relative abundance of specific microbes at various taxonomic levels can differ significantly [9,10,11,12,13,14,15,16,17,18]. While some differential microbes have been reported by different groups, the overlap between studies has been relatively low, highlighting the influence of both genetic and numerous environmental factors on the microbiome, such as population/ethnicity, geographic area, diet, culture, etc., limiting the ability to identify a global, specific MS microbiota signature. Some studies have addressed whether the gut microbiota is related to disease activity and progression [11,18,19,20,21,22] and, recently, whether disease-modifying therapies (DMTs) affect the microbiota or whether the microbiota may influence the efficacy or adverse events of DMTs [23,24,25,26,27,28,29]. Most of these studies compared the microbiota of untreated to treated patients in general, and while a few longitudinal studies have described the effect of a specific DMT on the microbiota, the vast majority have focused on the effects of dimethyl fumarate (DMF), perhaps due to its well-known gastrointestinal side effects [27,28,29,30,31,32]. Although DMTs were shown to modify microbial abundance, it remains unclear whether this is mainly an indirect result of immunomodulation and a general reduction in the inflammatory state of the immune system, including in the intestine, or whether it is through direct and specific drug−microbe interactions. In the first case, DMT−microbiota interactions could also be expected to reflect the degree of efficacy of a drug, with more pronounced effects seen for high-efficacy versus low-efficacy drugs. In the other case, the effects of a specific DMT could be expected to reflect the mode of action and drug administration route. The ongoing debate of escalation versus induction/high-efficacy treatment strategies for the best outcome and risk/benefit for patients [33,34], together with the potential role of the microbiota in disease course, further highlights the importance of elucidating DMT−microbiota interactions. In this study, we aimed to characterize the longitudinal effects of two DMTs with different modes of administration and action: Interferon beta-1a (INFβ-1a) (Rebif), as a subcutaneous injectable DMT with a moderate effect, and the high-efficacy oral DMT CladT (Mavenclad). Endogenous interferons mediate inflammatory responses to pathogenic stimuli, including in the gut [35], and treatment with IFN-β is thought to enhance anti-inflammatory, regulatory T cell responses [6]. Currently, there are three IFNβ subtypes available in clinical use for MS: IFNβ-1a, pegylated IFNβ-1a, and IFNβ-1b [6]. IFNβ treatment modifications of the microbiota in PwMS have been reported in a few cross-sectional studies or short-term longitudinal studies [24,31,36,37,38], but this is the first long-term longitudinal study on the effects of IFN-β-1a on the microbiota. Treatment with CladT introduces a transient immunodepletion of CD4^+^ and CD8^+^ T cells and CD19^+^ B cells, followed by partial reconstitution and prolonged immunomodulation [6]. Only one recent pilot study addressed the effect of CladT on the microbiome [39]. In this longitudinal study, we characterized INFβ-1a- and CladT-related modifications of the microbiota following 6 months of therapy, and we identified possible associations between the microbiota and clinical response. The evaluation of two DMTs under similar technical conditions within the same population and geographic region, consistent with our previous study of DMF [28], enabled cross-DMT comparisons across agents with distinct modes of action and efficacy, as well as differing cohort disease characteristics, thereby advancing our understanding of MS–DMT−microbiota interactions.

## 2. Results

### 2.1. Characteristics of Study Cohorts

Table 1 summarizes the demographic and clinical data of the participants. PwMS about to initiate treatment with IFNβ-1a (*N* = 31), or CladT (*N* = 30) were recruited to the study, providing a fecal sample at baseline and post-6 months from treatment initiation. Patients were followed clinically for 1 year (INFβ-1a) or 2 years CladT) post treatment initiation. At recruitment, 77% of the INFβ-1a cohort and 13% of the CladT cohort were treatment-naïve patients, while the remaining patients were DMT-free for 3.9 [1.0–9.1] and 2.3 [1.0–3.6] months, respectively. Mean disease duration was less than a year in the INFβ-1a cohort and 8.9 years in the CladT cohort. These differences between the two separate study cohorts reflect the differences in efficacy class of the two investigated drugs. The mean vitamin D level was >50 nmol/L in both cohorts. The Mediterranean diet score (MDS) was similar between pre- and post-6-month treatment visits in both cohorts, and only one INFβ-1a and two CladT patients were vegetarians. In the INFβ-1a cohort, 17 patients provided a second fecal sample after 6 months of therapy. During the 1-year follow-up period, five patients stopped INFβ-1a therapy due to intolerable adverse events (IAEs), and two patients were lost to follow-up. A total of 10 patients presented with clinical signs of disease activity (DA) within follow-up (of these, two patients presented with confirmed EDSS progression only, in the absence of relapse or MRI activity), while 14 patients remained NEDA according to the definition detailed in the Methods section. In the CladT cohort, 19 patients provided a second fecal sample after 6 months of therapy. During the 2-year follow-up period, five patients were lost to follow-up. A total of 15 patients achieved NEDA status after 1 year, and 7 maintained NEDA after 2 years, while 12 patients presented with DA within 1 year, increasing to 18 patients within 2 years (of these, 2 patients presented with confirmed EDSS progression only). Of the patients with DA, six patients switched to another DMT during the 2-year follow-up period due to an insufficient response. One patient received only the first treatment course of CladT due to unresolved lymphopenia. While there was no significant change in mean EDSS or MSSS over the 1-year follow-up period in the INFβ-1a cohort, mean MSSS was significantly reduced after 2 years of therapy compared to baseline in the CladT cohort (3.9 ± 0.6 vs. 5.6 ± 0.5, *p* = 0.041), while the reduction in mean EDSS was not statistically significant.

### 2.2. Microbiota Profiling of Patients Treated with INFβ-1a or CladT

#### 2.2.1. INFβ-1a Cohort

**Figure 1:**
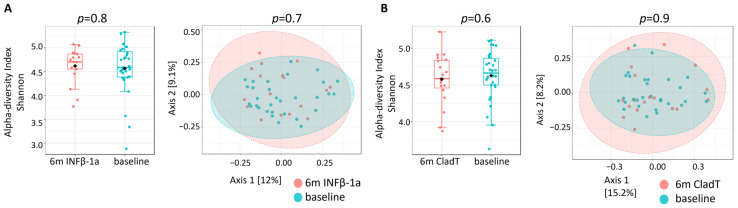
Comparison of general microbiome composition. (**A**)—INFβ-1a cohort and (**B**)—CladT cohort. (**Left**)—Shannon α-diversity (species level) of PwMS following 6-month therapy compared to pre-treatment (baseline). The line inside the box represents the median, and the black diamond dots represent the average of samples. (**Right**)—Bray–Curtis β-diversity (species level) of PwMS following 6 months of therapy compared to pre-treatment. Abbreviations: m—months.

There was no significant difference in alpha- or beta-diversity following INFβ-1a treatment (Figure 1A,B).

**Figure 2:**
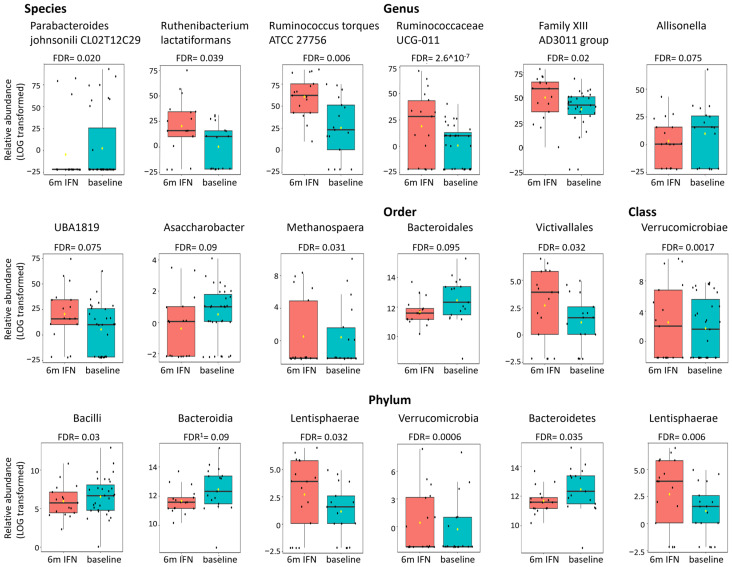
Representative graphs of microbes from Table 2A showing differential relative abundance following 6 months of INFβ-1a therapy compared to baseline. Significant by representative statistical method, either EdgeR or ^1^ DeSeq2. Each black dot represents the relative abundance of a participant sample. Boxes represent the median and interquartile range (IQR; 25th–75th percentiles). Abbreviations: FDR—false discovery rate, IFN—IFNβ-1a, m—months.

Table 2A and Figure 2 present the microbes that exhibited significant differential abundance following 6 months of INFβ-1a therapy compared to baseline (results of all analyses are presented in Table S1A). The phyla Verrucomicrobia and Lentispaerae were more abundant after 6 months of INFβ-1a therapy, while Bacteroidetes were less abundant. Similarly, the classes Verrucomicrobiae and Lentispareria were more abundant, while Bacteroidia and Bacilli were less abundant following INFβ-1a therapy. On the order level, Bacteroidales was less abundant, while Victivallales was more abundant following treatment. Seven genera exhibited significant differential abundance following INFβ-1a therapy; *Ruminococaceae UCG-011*, *Family XIII AD3011 group*, *Kleibsiella*, *UBA1819* and *Methanosphaera* were enriched, while *Allisonella* and *Asaccharobacter* were reduced. Six species differed significantly following INFβ-1a therapy; *Ruminococcus torques ATCC 27756 Ruthenibacterium lactatiformans*, and three not fully defined species were enriched, while *Parabacteroides johnsonii CL02T12C29* was reduced.

#### 2.2.2. CladT Cohort

**Figure 3:**
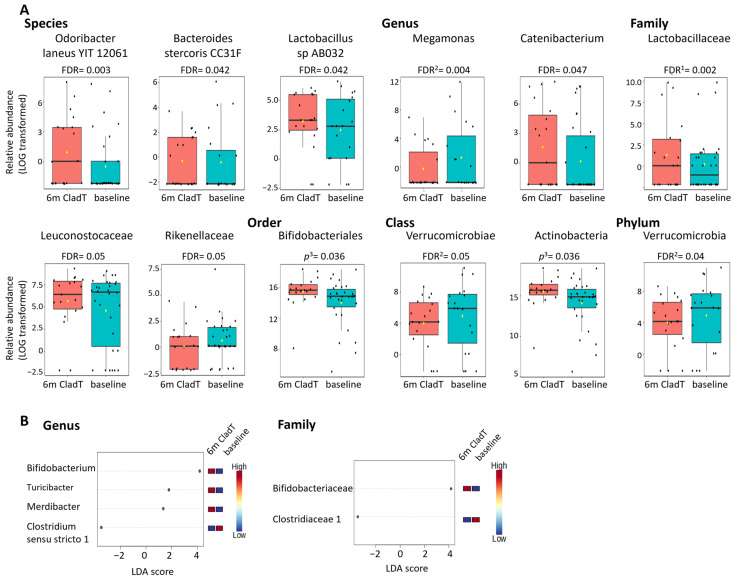
Representative graphs of microbes from Table 2B exhibiting differential relative abundance following 6 months of CladT therapy compared to baseline. (**A**)—Representative significant statistical method: MetagenomeSeq or ^1^ DeSeq2, ^2^ EdgeR and ^3^ LEfSe and (**B**)—LEfSe. In A, each black dot represents the relative abundance of a participant sample, and boxes represent the median and interquartile range (IQR; 25th–75th percentiles). Abbreviations: FDR—false discovery rate, LDA—linear discriminant analysis, m—months, *p*—*p*-value.

There was no significant difference in alpha- or beta-diversity following CladT treatment (Figure 1B). Table 2B and Figure 3 present the microbes that exhibited significant differential abundance following 6 months of CladT therapy compared to pre-treatment (results of all analyses are presented in Table S1B). The relative abundance of phylum Verrucomicrobia and class Verrucomicrobiae was reduced, while the abundance of class Actinobacter and order Bifidobacteriales was elevated following CladT therapy. Two families were enriched (*Lactobacillaceae* and *Bifidobacteriaceae*), while *Leuconostocaceae* and *Rikenellaceae* were reduced. Four genera were enriched (*Bifidobacterium*, *Merdibacter*, *Catenibacterium* and *Turicibacter)*, while three families, *Enterobacter*, *Megamonas* and *Clostridium sensu stricto 1*, were reduced following therapy. Moreover, five species were enriched following CladT: *Odoribacter laneus YIT 12061*, *Bacteroides stercoris CC31F*, *Lactobacillus* sp. *AB032*, and two not fully defined species.

### 2.3. Associations Between Microbiota Profile and Clinical Response

In order to identify possible associations between specific microbe abundance and response to therapy, we compared the relative abundance in samples obtained pre-treatment or following 6 months of INFβ-1a or CladT treatment between patients who remained NEDA and patients who developed DA (relapse, MRI activity and/or confirmed disability progression) after 1 year (INFβ-1a) or after 1 and 2 years (CladT) of clinical follow-up.

#### 2.3.1. Associations in the INFβ-1a Cohort

Table 3 presents the microbes that exhibited significant differential abundance according to disease activity status in INFβ-1a-treated patients at the 1-year follow-up (Tables S2A,B present all results). In pre-treatment samples, one species (*Bacteroides thetaiotaomicron*) and two genera (*Alloprevotella* and *Anaerostipes*) were more abundant in patients who remained NEDA after 1 year of therapy, while two species, one genus (*Mitsuokella*), two orders (Gastranaerophilales and Rhodospirillales), two classes (Melainabacteria and Alphaproteobacteria), and one phylum (Cynobacteria) were more abundant in patients who developed DA during 1-year follow-up. In samples obtained following 6 months of INFβ-1a therapy, two genera (*Enterococcus* and *Klebsiella*), the *Enterococcaceae* family, and the Aeronadales order were more abundant in DA patients (according to at least two methods); notably, these taxa differed from those identified in pre-treatment samples.

#### 2.3.2. Associations in the CladT Cohort

Table 4 and Table 5 present the microbes that exhibited significant differential abundance according to disease activity status at the end of 1- and 2-year follow-up, respectively, in CladT-treated patients (all results are presented in Table S3A–D). In samples obtained at baseline, three species were more frequent in patients who remained NEDA after 1 year, and four species were more frequent in patients remaining NEDA after 2 years, with *Acidaminococcus intestine DSM 21505* and *Bifidobacterium* sp. *MC_10* appearing in both analyses. Six genera were more frequent, and one was less frequent in patients who remained NEDA after 1 year, while three different genera were more frequent and two were less frequent in patients remaining NEDA after 2 years. Five families were more abundant in patients who remained NEDA after 1 year, such as *Methanobacteriaceae* and *Synergistaceae*, while *Burkholderiaceae* was less frequent. The orders Methanobacteriales and Opitutales were more abundant, while Betaproteobacteriales was less abundant in patients who remained NEDA after both 1 and 2 years, and two additional orders (Pasteurellales and Verrucomicrobiales) were significantly associated with NEDA after 1 year only. The class Methanobacteria was associated with NEDA after 1 year, while Gammaproteobacteria was associated with DA after 2 years. Finally, Euryarchaeota and Verrucomicrobia were more abundant in patients remaining NEDA after 1 year, while Euryarchaeota and Cyanobacteria were associated with NEDA and Proteobacteria associated with DA after 2 years. Analysis of microbe abundance in samples obtained following 6 months of CladT therapy identified seven species with differential abundance between patients with NEDA and those with DA at 1 year and five species at 2 years, with *uncultured archaeon* and *Lactobacillus* sp. *ABO32* overlapping between both analyses. A total of 11 genera were differential according to NEDA status following 1 year, and 10 genera following 2 years, with *Methanobrevibacter* and *Eisenbergiella* overlapping between analyses and both associated with NEDA. The family *Methanobacteriaceae* was more abundant in patients remaining NEDA after both 1 and 2 years of therapy, while four additional families were associated with NEDA after 2 years. The orders Methanobacteriales and Synergistales were more abundant in patients remaining NEDA after both 1 and 2 years of therapy, while Opitutales was associated with NEDA after 2 years, and Betaproteobacteriales was associated with DA after 1 year. The class Methanobacteria was associated with NEDA after both 1 and 2 years of therapy; at 2-year follow-up, Synergistia was more abundant, while Mollicutes was less abundant in patients remaining NEDA. Finally, the phylum Euryarchaeota was more abundant in patients remaining NEDA after both 1 and 2 years of therapy; at the 2-year follow-up, Synergistetes was more abundant, while Tenericutes was less abundant in the NEDA group. Microbes that exhibited a similar association with disease activity in samples obtained at baseline or post-6 months CladT therapy included *Methanobrevibacter*, *Methanobacteriaceae*, Methanobacteriales, Methanobacteria and Euryarchaeota, all of which were more abundant in patients remaining NEDA after 1 year. Furthermore, *Acidaminococcus intestine DSM 21505*, Methanobacteriales, Opitutales and Euryarchaeota were associated with NEDA, while Betaproteobacteriales was associated with DA at the 2-year follow-up.

#### 2.3.3. Correlation Between Microbe Abundance and Change (Δ) in EDSS or MSSS Scores

**Figure 4:**
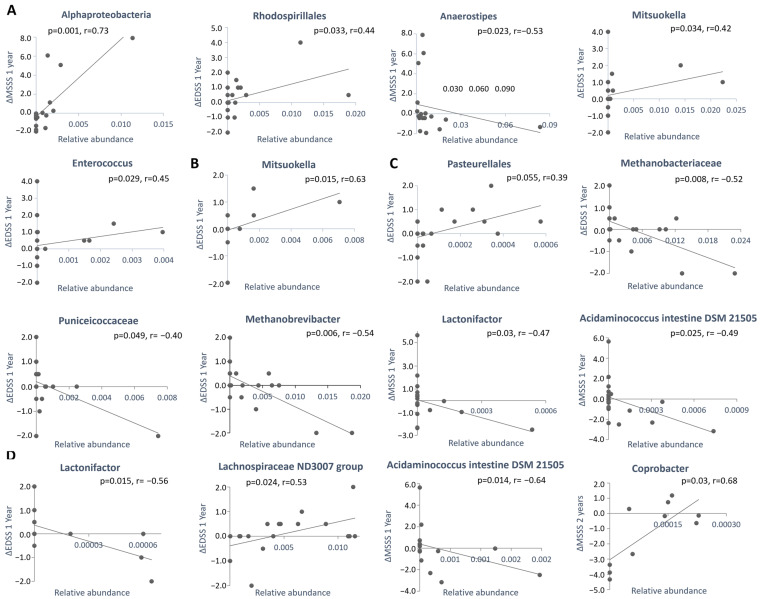
Representative correlations between microbial abundance and clinical progression (from Table 6A,B). Spearman’s rank correlation was computed between the relative abundance of microbes associated with NEDA or disease activity (as identified in Table 4, Table 5 and Table 6) and ΔEDSS or ΔMSSS at 1 or 2 years. Panels represent (**A**) baseline samples from the IFNβ cohort; (**B**) samples obtained after 6 months of IFNβ therapy; (**C**) baseline samples from the CladT cohort; (**D**) samples obtained after 6 months of CladT therapy. Abbreviations: r—correlation coefficient.

To further explore the associations between microbial abundance and disease activity, Spearman’s rank correlation coefficients were calculated between the relative abundance of significantly differentially abundant microbes (at baseline or following 6 months of therapy) and the longitudinal changes (Δ) in EDSS or MSSS scores at 1 and 2 years. Significant correlations are presented in Table 6A,B (IFNβ and CladT), and representative graphs are shown in Figure 4. The identified correlations were moderate. In the IFNβ cohort, NEDA-associated *Anaerostipes* correlated negatively with ΔEDSS and ΔMSSS, while NEDA-associated *Bacteroides thetaiotaomicron* correlated negatively with ΔEDSS. Conversely, DA-associated Alphaproteobacteria and Rhodospirillales correlated positively with both ΔEDSS and ΔMSSS. In the CladT cohort, identified correlations included NEDA-associated Euryarchaeota, *Methanobacteriaceae* and *Methanobrevibacter*, which correlated negatively with ΔEDSS at both 1 and 2 years and with ΔMSSS at 1 year. Additionally, NEDA-associated *Lactonifactor* correlated negatively with ΔEDSS and ΔMSSS in both baseline and 6-month samples. Conversely, DA-associated Pasteurellaes correlated positively with ΔEDSS after 1 years, while DA-associated *Coprobacter* correlated positively with both ΔEDSS and ΔMSSS after 2 years.

### 2.4. Associations Between Microbiota Profile and Intolerable Adverse Events in INFβ-1a-Treated Patients

We compared the baseline microbial relative abundance between patients who developed IAE leading to discontinuation of INFβ-1a therapy and those who tolerated and continued therapy throughout the 1-year follow-up (Table S4). Significant differential abundance was observed for 9 species, 12 genera, 4 families, 2 orders and 1 class between patients with and without IAE. Specifically, species *Parabacteroides johnsonii CLO2T12C2* and *Bifidobacterium* sp. *MC_10*, genera *Negativibacillus*, *Sarcina*, *Tyzzerella 4* and *Lactobacillus*, families Flavobacteriaceae and Puniceicoccaceae, and class Bacilli were associated with the occurrence of IAE, whereas *Bacteroides thetaiotaomicron* and *Alloprevotella* were associated with the absence of IAE.

### 2.5. Functional Metabolic Pathway Analysis

Enrichment of functional metabolic pathways in samples after 6 months of INFβ-1a or CladT therapy (compared to baseline) and in NEDA patients (compared to those with DA) following 1 or 2 years of therapy was predicted using Tax4Fun, based on the Kyoto Encyclopedia of Genes and Genomes (KEGG) database [40,41] (Table 7). LEfSe analysis identified 10 significant KEGG orthologies (KOs) that distinguished between samples obtained post-6-month INFβ-1a from those at baseline, which mapped to two enriched pathways, namely glycosaminoglycan degradation and drug metabolism (Table 7A(1)). Using a univariate analysis, we identified 26 KOs that differed significantly in samples post-6-month INFβ-1a, which mapped to 6 additional enriched pathways (Table 7A(1)), including pathways of ubiquinone biosynthesis, amino acid degradation and the citrate cycle. We identified 125 KOs that significantly distinguished between patients remaining NEDA after 1 year of INFβ-1a therapy and those with DA, which mapped to 13 enriched functional pathways, including fructose and mannose metabolism, galactose metabolism, fatty acid degradation and amino acid degradation (Table 7A(2)). In the CladT cohort, LEfSE analysis revealed 52 significant KOs that distinguished between samples obtained post-6-month CladT from those at baseline, mapping to 3 enriched pathways, including propanoate and galactose metabolism (Table 7B(1)). There were 171 significant KOs that distinguished between patients remaining NEDA after 1 year of CladT therapy and those with DA, mapping to 8 enriched pathways, such as amino acid and fatty acid degradation, butanoate metabolism, and methane metabolism (Table 7B(2)), while 108 KOs were significant according to NEDA status, following 2 years of CladT therapy, mapping to 2 enriched pathways, namely methane metabolism and pantothenate and CoA biosynthesis (Table 7B(3)).

### 2.6. Association Between Clinical Response and Adherence to a Mediterranean Diet or Specific Nutrient Intake

There was no significant difference in MDS of patients at recruitment between those who developed DA and those remaining NEDA after 1 year of INFβ-1a therapy or remaining NEDA after 1 or 2 years of CladT therapy (Table S5). There was also no baseline difference in MDS between patients who developed IAE leading to discontinuation of INFβ-1a therapy and those who tolerated and continued therapy throughout follow-up (7.0 + 1.3 vs. 6.4 + 0.6, *p* = 0.6). We also compared the daily intake of energy and 78 various nutrients, calculated from a Food Frequency questionnaire at baseline, between patients who maintained NEDA status and those who developed DA following 1 year of INFβ-1a therapy or 1 and 2 years of CladT therapy (significant results are presented in Table S6). There were no significant differences between groups for most nutrients. However, in the INFβ-1a cohort, the intake of fructose was significantly higher in patients who developed DA than in those with NEDA. In the CladT cohort, patients with DA at the 1-year follow-up exhibited a higher baseline intake of trans fatty acids, a higher caloric percentage from fat and saturated fat, and a lower caloric percentage from carbohydrates compared to NEDA patients. Moreover, baseline alcohol intake was higher in CladT patients who maintained NEDA status at the 2-year follow-up compared to those who developed DA. Similarly, we compared the baseline mean daily nutrient intake between patients who developed IAE following INFβ-1a initiation and those who continued INFβ-1a treatment throughout the follow-up period (significant results are presented in Table S7). The mean intake of sodium, iron and sugar was significantly lower in patients who developed IAE. Additionally, statistical trends toward lower intake were observed for total food energy and nutrients such as proteins, carbohydrates, and several amino acids and minerals.

## 3. Discussion

In this longitudinal study, we have identified significant DMT-associated modifications of the gut microbiota following 6 months of INFβ-1a or CladT therapy and have explored potential associations between microbiota and clinical response. Due to the genetic, environmental, and lifestyle factors that influence the specific microbiota of an individual, a healthy gut microbiota is in general defined as a balanced, high-diversity and resilient microbiota composition, and it is important for the proper immune functions of the host and prevention of disease. There are accumulating reports of differentially abundant gut microbes in PwMS compared to healthy individuals [9,10,11,12,13,14,18], but there has been relatively low uniformity across studies, emphasizing the impact of multiple factors affecting microbiota composition, such as genetic heterogeneity, culture, diet, and also technical methods of microbial DNA analysis. A few reports have described associations between the relative abundance of specific microbes and MS disease activity or progression [11,18,19,20,21,22]. While these reports are mainly descriptive and not causative, experimental studies using experimental autoimmune encephalomyelitis (EAE) mice models have provided evidence-based indications that the microbiota is capable of influencing both MS-like development and progression [42,43,44]. Moreover, EAE models have shown that microbiota-directed interventions, including antibiotics, probiotics, and fecal transplantation, possess the potential to modulate disease symptoms and clinical course [45,46,47]. Several studies have described MS-DMT-associated alterations in microbial abundance [24,25,26,27,28,29], suggesting that the beneficial therapeutic effects of these agents may be partially mediated by modifications of the gut microbiota profile [31]. Microbe–drug interactions may include direct effects on pharmacokinetics, through mechanisms such as biodegradation or drug activation/deactivation and transformation of drugs into secondary metabolites, or may be indirect, through microbial metabolites that can affect signaling pathways and metabolism of the host or may compete with drug receptors [23,48]. There are several examples of how drug response may vary according to microbiota composition. For example, the cardiac drug digoxin is inactivated by the microbe *Eggerthella lenta*, while the efficacy of the chemotherapeutic agent cyclophosphamide is enhanced in the presence of *L. Johnsonii.* Furthermore, gemcitabine, another chemotherapeutic agent, is inactivated by an enzyme present in Gammaproteobacteria, leading to drug resistance; meanwhile, the anti-tumor effect of the immunotherapy drug Ipilimumab (targeting CTLA-4) depends upon the presence of *Bacteroides thetaiotaomicron* and *Bacteroides fragilis* in the gut [48,49,50].

In this study, we focused upon characterizing the effects of INFβ-1a and CladT therapy on the gut microbiota; they are two DMTs with distinct modes of administration, mechanisms of action, and degrees of efficacy, which have scarcely been investigated in the context of the microbiota. A few studies have shown that INFβ therapy can modify the gut microbiota in PwMS [24,31,36,37,38]; however, these have mostly been cross-sectional studies or studies of short (2-month) duration. In a cross-sectional study of 15 INFβ-1b-treated compared to 15 untreated patients, there was no difference in α or β-diversity or in the abundance of specific phyla, and the only significant difference found was a higher abundance of *Prevotella copri* (phylum Bacteroidetes) in treated patients [37]. In another cross-sectional study focused upon the abundance of Firmicutes members in 39 IFNβ-1-treated versus 25 untreated patients, no therapy-induced differences were found; however, some sex-specific associations between IFNβ and microbial abundance were observed [36]. In a short-term longitudinal study of 11 IFNβ-1a-treated patients assessed before and after 2 months of therapy, the overall diversity was not affected by therapy, and the only significant differences were reduced abundance of *Lachnospiraceae*, while increased abundance of *Peredibacter* (phylum Bdellovibrionota) following therapy [38]. In another short-term longitudinal study of 10 IFNβ-1a-treated patients followed for 8 weeks, no significant IFNβ-associated differences in microbial abundance were reported [24]. In a large cross-sectional study by the iMSMS consortium comparing 87 IFN-treated and 209 untreated PwMS, the β-diversity of the microbiota in the IFNβ-treated cohort differed significantly. Furthermore, IFN treatment was associated with a reduced abundance of *Clostridium* sp. *CAG:91* (family *Clostridiaceae*), *Dialister invisus* (family *Veillonellaceae*), and *Butyrivibrio crossotus* (family *Lachnospiraceae*) and an increased abundance of *Bacteroides coprophilus* (family *Bacteroidaceae*) [31]. In the present longitudinal study, we found no significant effect of IFNβ on the α- or β-diversity of the microbiota; however, we identified several microbes from various phyla that were modified following IFNβ therapy. In general, the phylum Bacteroidetes and several of its members, such as Bacteroidia and Bacteroidales, were reduced following IFN therapy. In contrast, members of Firmicutes were elevated following therapy, such as several microbes from the family *Lachnospiraceae* and *Ruthenibacterium lactatiforman* from the family *Ruminococcaceae*. Moreover, members of the class Verrucomicrobiae as well as *Methanosphaera* (phylum Euryarchaeota) were elevated following IFNβ therapy. These results did not overlap with results from previous cross-sectional or short-term longitudinal studies, most likely due to the different study design (cross-sectional versus longitudinal study), short study period (2 versus 6 months), technical differences, and generally small cohort sizes encumbering the prospect of reaching statistical significance. In a previous longitudinal study [28], we found that the relative abundances of Bacteroidetes, Bacteroidia and Bacteroidetes were reduced following six months of DMF therapy, similar to the effects observed with INFβ-1a therapy. Others have found that Bacteroidetes abundance decreases following 3 months of DMF therapy [29], while *Bacteroides* has been reported to decline following both DMF and ocrelizumab therapy [29,51]. *Ruthenibacterium lactatiforman*, an SCFA-producing microbe, was similarly elevated in patients treated with fingolimod and glatiramer acetate [31] as well as in those receiving natalizumab [52], consistent with our current observations following IFNβ. Additionally, Verrucomicrobia was similarly elevated in DMT-treated versus untreated pediatric MS patients, where 10 out of 23 DMT-patients were treated with IFNβ [53]. The crosstalk between the gut microbiota, SCFAs, and T cells plays a key role in the maintenance of immune responses. Several gut microbes produce SCFAs, most of which belong to the phyla Actinomycetota, Bacteroidetes, Firmicutes and Proteobacteria [54]. SCFAs exert significant anti-inflammatory effects by promoting T regulatory cell (Treg) differentiation and anti-inflammatory IL-10 cytokine production, while reducing inflammatory Th17 cell and IL17 production, thereby restoring the Treg/Th17 imbalance. Moreover, SFCAs strengthen the integrity of the intestinal epithelial barrier by stimulating mucin production and enhancing tight junctions within the intestinal epithelium; they also contribute to intestinal homeostasis, function, and energy production. SCFAs also support the integrity of the blood–brain barrier (BBB) and play important immunomodulatory roles within the CNS [55,56,57,58]. Firmicutes are important producers of butyrate, with the *Lachnospiraceae*, *Lactobacillaceae* and *Rumanococcaceae* families being the primary contributors. Thus, the IFN-related enrichment of the *Lachnospiraceae* and *Ruminococcaceae* families observed in this study indicates a beneficial increase in SCFA-producing potential. In general, the intestinal epithelial and the mucosal immune system are in ongoing contact with dietary- and gut microbiota-derived antigens, which trigger endogenous secretion of type 1 interferon that mediates local innate and adaptive immune responses, particularly affecting dendritic and T cells [35,59,60]. IFNβ, a member of the type 1 interferon family, mediates local intestinal responses to viral and bacterial pathogens, as well as commensal microbiota. It acts by stimulating the production of anti-inflammatory cytokines (such as IL-10), promoting Treg proliferation and regulatory function, and inducing suppressive B cells, while simultaneously reducing pro-inflammatory cytokines, B cell antigen-presenting capacity, and overall inflammation [35,61,62]. IFNβ was shown in a mice model to upregulate tight junction proteins in lung epithelial cells and in blood vessel endothelial cells and shown to reduce BBB permeability and T cell transmigration in in vitro models [35,63,64,65]. Given the established role of endogenous IFNβ in the intestine, the results of the present study support that the mode of action of IFNβ therapy in PwMS involves the modification of the microbiota and the enrichment of microbiota-derived compounds, such as SCFAs. Interestingly, a study found that IFNβ-treated PwMS had significantly higher serum levels of the SCFA propionate, and the authors suggested that IFNβ may increase the absorption of propionate by upregulating the transporter of SCFA (monocarboxylate transporter-1) [31], thereby suggesting an additional route by which IFNβ–microbiota–SFCAs may interact. However, in a study of 23 PwMS, serum levels of SCFAs and of medium-chain fatty acids (MCFAs) (such as caproic acid)—which promote inflammatory processes and increase Th1/Th17 cells—did not differ significantly from baseline following one year of therapy [66].

In the present study, we found that the oral drug CladT, with a unique short course of administration of tablets taken over two weeks per year for two years only, induces modifications to the gut microbiota that are detectable 6 months from the initiation of therapy. In the only prior publication addressing CladT-mediated effects on the microbiome—a 12-month longitudinal study of 25 RRMS patients—no significant changes in α-diversity or in microbial abundance at the phyla or species level were observed [39]. Similar to reports from other DMTs, we found that CladT did not affect the α- or β-diversity of the microbiota. In contrast to IFNβ, the abundance of Verrucomicrobia was reduced following CladT therapy. However, consistent with the patterns observed for IFNβ, several members of the Firmicutes phylum were elevated; these included genera from the *Erysipelotrichaceae* family (such as *Catenibacterium)*, the *Lactobacillaceae* family, members of the *Lachnospiraceae* family, a *Faecalibacterium* species within the *Ruminococcaceae* family, *Lactobacillus* sp. *AB032* within the *Veillonellaceae* family, and a *Lactobacillus* species belonging to the *Lactobacillaceae* family. Several members of the Actinobacteria phylum, such as *Bifidobacteriaceae and Bifidobacterium*, were also enriched following CladT therapy. In contrast, members of Proteobacteria and of the *Clostridiaceae* family were reduced following CladT, including the potential opportunistic pathogen *Clostridium sensu stricto 1.* Furthermore, within the Bacteroidetes phylum, divergent trends were observed: some members, such as *Rikenellaceae*, were reduced, whereas others, like the *Muribaculaceae* family, were increased. Thus, CladT therapy, similar to IFNβ, is associated with the modification of several microbial taxa and the enrichment of beneficial SCFA-producers, such as *Lachnospiraceae*, *Lactobacillaceae* and *Rumanococcaceae* members. *Bifidobacterium* is a well-established and widely utilized probiotic recognized for its beneficial effects on the intestinal mucus layer, barrier integrity, and health in general [55,67,68]; reduced abundance of Bifidobacterium has been demonstrated in various diseases [4]. A lower *Bifidobacterium* to *Akkermansia* ratio was associated with disease and disease severity in an EAE model, and a lower *Bifidobacterium adolescentis* to *Akkermansia muciniphila* ratio was associated with MS and with a higher EDSS score in PwMS [31,69]. CladT also increased the abundance of an undefined species from *Faecalibacterium*, another beneficial SCFA producer and indicator of intestinal health that has been shown to be less abundant in PwMS than healthy controls [17]. Interestingly, we previously demonstrated that, similar to CladT, six months of DMF therapy also reduced the abundance of *Leuconostocaceae* and *Rikenellaceae* [28]. Cladribine (2-chlorodeoxyadenosine) is a synthetic deoxyadenosine analogue that causes selective, sustained lymphocyte depletion in PwMS. This action depends upon the relative levels of enzymatic activity in the immune cells of deoxycytidine kinase (DCK), which phosphorylates cladribine to its active metabolite (Cd-ATP), and 5′-nucleotidase, which degrades Cd-ATP. Cladribine affects both dividing and resting cells by impairing DNA synthesis and inducing cell death, and this process ultimately leads to immune reconstitution characterized by long-term immunological shifts [70,71,72]. Beyond its role in immune repletion, cladribine is thought to modulate the cytokine milieu and T cell migration [73,74]; our data extend these observations by revealing a novel impact on gut microbiota composition.

Deciphering whether the microbiota can influence the clinical response to a DMT is of utmost importance. Besides providing predictive value, such insight could lead to development of microbiota-based interventions (such as pre-, pro- and/or post-biotics) that may ultimately improve DMT efficacy. While probiotics were shown in preclinical studies to beneficially reduce pro-inflammatory markers and induce anti-inflammatory responses, to ameliorate disease severity and progression in EAE and MS, and to improve mental health of PwMS [46,55,75,76], it remains to be determined if probiotics may synergistically improve the clinical outcome of a DMT. In both baseline and 6-month-post-treatment samples, a higher abundance of various members of Bacilli and Proteobacteria (including Rhodospirillales) was associated with DA within one year of IFNβ therapy, whereas a higher abundance of Cyanobacteria members was associated with DA at baseline only. In contrast, a higher abundance at baseline of butyrate-producing *Anaerostipes* (from the *Lachnospiraceae* family) and of *Bacteroides thetaiotaomicron* and *Alloprevotella* was associated with NEDA status. The association between microbial abundance and disease activity was further supported by identified negative correlations between the abundance of NEDA-associated microbes and ΔEDSS or ΔMSSS; in contrast, these clinical metrics correlated positively with the abundance of DA-associated microbes. *Bacteroides thetaiotaomicron* is a prominent commensal gut microbe that degrades both dietary-derived and host-derived polysaccharides—including of the intestinal mucus layer—to produce the SCFA acetate [77] as well as polyamines, which may diminish neuroinflammation [55]. The beneficial role of this microbe was supported by its significant association with both the achievement of NEDA and the absence of IAE in IFNβ-treated patients. Interestingly, a recent study identified the baseline level of Rhodospirillales as a robust contributor to a model predicting MS disease worsening. That same study found that *Alloprevotella* and Rhodospirillales were positively correlated with disease progression and EDSS increase, whereas *Akkermansia* and SCFA-producers such as *Lachnospiraceae* exhibited negative correlations with disease worsening [19].

Among CladT-treated patients, baseline analysis revealed that a higher relative abundance of several microbes—including *Bifidobacterium* sp. *MC_10* (Actinobacteria), members of Firmicutes (such as *Lachnospiraceae* family members), *Streptococcus salivarius subsp. thermophilus* (order Lactobacillales), and *Akkermansia* (phylum Verrucomicrobia)—was associated with NEDA status at one and/or two years of follow-up. In contrast, a higher abundance of several members of Proteobacteria was associated with DA within the same timeframe. Exploratory analysis of the microbiota following six months of therapy revealed consistent correlations with clinical outcome. Specifically, a higher abundance of Euryarchaeota members (such as *Methanosphaera)*, Opitutales (Verrucomicrobioa), several Firmicutes members (including *Lachnospiraceae* family members), *Ruthenibacterium lactatiformans*, *Lactobacillus* sp. *AB032*, *Streptococcus salivarius subsp. thermophilus* and *Catenibacterium*, along with *Prevotella* and *Massiliprevotella massiliensis* from the Bacteroidetes phylum was observed in patients remaining NEDA after one and/or two years. Conversely, Bacteroidetes members, including *Bacteroides ovatus V975* and Proteobacteria members, were associated with DA. Many of these associations between microbial abundance and NEDA status were further supported by identified correlations between microbe abundance and ΔEDSS and/or ΔMSSS following 1–2 years of therapy. Consistent with our current findings for CladT, in our previous study on DMF [28], a higher abundance of *Massiliprevotella massiliensis* and *Catenibacterium* was associated with NEDA one year following DMF initiation; conversely, a higher abundance of Proteobacteria and *Bacteroides ovatus V975* was linked to DA. The *Prevotella* genus was found to be reduced in PwMS compared to healthy controls across several studies [9,11,18,78], while its abundance appeared to increase following treatment [9], suggesting a potentially beneficial role mediated by a reduction in oxidative stress and inflammatory cytokines [55]. However, other reports have paradoxically associated *Prevotella* with MS disease worsening [19]. Cox et al. [52] reported that *Akkermansia* correlates negatively with EDSS and MRI lesion burden in PwMS. Furthermore, they showed that *Akkermansia* strains isolated from PwMS can ameliorate disease in an EAE model, suggesting a beneficial protective role for *Akkermansia* in MS, supporting the association observed in our study between higher levels of *Akkermansia* and NEDA status in CladT-treated patients. Integrating the data on microbial shifts following CladT therapy with clinical outcomes identifies a subset of microbes of particular interest: these microbes were not only enriched by CladT therapy but their higher abundance at baseline and/or after 6 months of therapy was also associated with sustained NEDA status over a 1–2 year period, namely *Muribaculaceae*, *Catenibacterium*, *Lactobacillus* sp. *AB032* and *Bifidobacterium* sp. *MC_10*. The *Muribaculaceae* family metabolizes mucin glycans and dietary fibers; produces SCFAs; has been shown to have mutualistic cross-feeding relationships with *Bifidobacterium* and *Lactobacillus*; and was found to be depleted in several autoimmune diseases [79]. *Lactobacillus* species are essential probiotic bacteria that balance the integrity of the intestinal and mucosal barriers. They inhibit inflammation by upregulating the induction of Tregs, suppressing Th1 and Th17 responses, and modulating the Th1/Th2 ratio. Furthermore, these species may influence the M1/M2 macrophage ratio and regulate oxidative stress responses within the gut [80,81]. Administration of probiotics consisting of three *Lactobacillus* species, one *Bifidobacterium* species and *Streptococcus thermophilus* to EAE mice suppressed EAE development and delayed disease progression. This was mediated by an increase in Tregs and a reduction in pro-inflammatory T cells [82]. Multiple studies using the EAE model have shown that probiotic supplementation can increase the production of anti-inflammatory cytokines IL-10, IL-4, and TGF-β and promote the differentiation of Tregs, while simultaneously reducing the levels of inflammatory cytokines and Th1/Th17 cells [46,75,83]. Moreover, such supplementation can increase the relative abundance of Firmicutes, *Bifidobacterium*, *Lactobacillus* and *Prevotella*, among other taxa. Consistently, supplementation with *Bifidobacterium* species has been associated with favorable clinical outcomes in the EAE model [84]. In humans, LBS probiotic supplementation (*Lactobacillus*, *Bifidobacterium* and *Streptococcus*) for two months increased the abundance of taxa depleted in PwMS and was associated with lower expression of HLA-DR on dendritic cells. Moreover, the abundance of *Lactobacillus*, *Streptococcus* and *Bifidobacterium* correlated negatively with the expression of HLA-DR on dendritic cells and of the MS risk allele HLA.DPB1 on monocytes [85]. In a recent study linking disease progression with microbiota abundance, three Bacteroides species were associated with a two-year increase in EDSS, while *Streptococcus thermophilus*, among others, was associated with an EDSS decrease [20]. Patients who experienced clinical worsening also exhibited decreased levels of *Streptococcus* and multiple *Lachnospiraceae* members alongside elevated levels of Bacteroides; these findings support the associations observed between these microbes and NEDA status in CladT-treated patients.

Baseline analysis identified several microbes with significantly different abundances between patients who discontinued IFNβ due to IAE and those who continued therapy throughout the follow-up period. However, as only 5 out of the 31 patients who initiated IFNβ experienced IAE, these findings must be considered highly exploratory. The main adverse events of IFNβ treatment are flu-like symptoms (FLSs), such as myalgia, arthralgia, fever and headache, occurring in more than half of PwMS initiating IFNβ treatment. While in many cases IFNβ-induced FLSs diminish over time, some patients experience persistent and intolerable FLSs, leading to drug discontinuation [86,87,88]. The mechanism underlying IFNβ-induced FLSs is unclear, and a literature search revealed no publications linking IFNβ-induced FLSs with the microbiota. The differentially abundant microbes identified in this study are generally characterized as commensal gut microbes. However, it is worth considering whether variations in microbial immunogenicity could explain why certain taxa are associated with an increased risk of IFNβ-related IAE. Moreover, the gut microbiota and their metabolites, such as SCFAs, can modulate IFN responses by promoting or suppressing IFN signaling pathways. The necessity of the gut microbiota for IFNβ responses has been demonstrated in germ-free or microbiota-depleted mouse models, where such responses were significantly attenuated [60]. These findings support that the microbiota may influence both the incidence of adverse events to IFNβ and the efficacy of IFNβ treatment.

All microbes perform in general four main metabolic pathways: biosynthesis, degradation, energy metabolism, and macromolecule modification [31]. In the current study, IFNβ therapy was associated with several enriched pathways, including amino acid degradation, biosynthesis of various molecules, and energy metabolism through the citrate cycle. In a previous study by the iMSMS consortium, IFNβ therapy was associated with an enrichment in L-ornithine biosynthesis, Heme synthesis and unsaturated fatty acid biosynthesis, along with a reduction in polysaccharide degradation. Moreover, our functional pathway predictions—while based on bioinformatic inference only—are partially corroborated by a metabolomic analysis of 23 IFNβ-associated stool metabolites from a cohort of 49 IFN-treated and 79 untreated patients. This analysis demonstrated an enrichment of the citrate cycle, consistent with our results, as well as of G-glutamine and D-glutamate metabolism, alongside amino acids metabolism and biosynthesis, purine, butanoate and nitrogen metabolism [31]. CladT therapy was associated with an enrichment of the functional pathways of propanoate and galactose metabolism, supporting that CladT enhances the level of beneficial SCFA propionate. In our previous study of DMF-treated patients [28], the citrate cycle and the propanoate metabolism were similarly enriched following DMF therapy. Several functional pathways were associated with NEDA in both IFNβ- and CladT-treated patients, including valine, leucine and isoleucine degradation, butanoate metabolism and fatty acid degradation. Furthermore, propanoate metabolism was enriched in CladT-treated patients maintaining NEDA status, further supporting a beneficial effect of both DMTs on SCFA production. Whether any of these functional pathway enrichments are of biological relevance awaits experimental validation.

Sustained dietary patterns can influence microbiota composition and affect health and disease [55,75]. Increasing the diet quality of PwMS is associated with a reduction in the risk of higher disability level and symptoms and with better physical and mental quality of life [89]. This may be linked to the effects of diet on oxidative stress, and subsequently on inflammation and neuronal damage, as well as the mitigation of vascular comorbidities associated with increased risk of disease activity and progression. Furthermore, these outcomes are likely influenced by diet–microbiome interactions that modulate the composition and function of the gut microbiota [75,89,90]. The Mediterranean diet, characterizing the Israeli population, is known to reduce risk of cardiovascular and neurodegenerative diseases and has demonstrated beneficial potential to reduce MS symptoms and EDSS scores in pilot studies [75,91]. However, in the current study, differences in adherence to a Mediterranean diet were not associated with the clinical response in either IFNβ- or CladT-treated patients, and only a few nutrients significantly differed between patients with NEDA status and those with DA. Among IFNβ-treated patients, a higher intake of fructose was positively associated with DA. Among CladT-treated patients, DA was associated with higher intake of trans fatty acids and a high percentage of calories from total and saturated fat alongside a low percentage of calories from carbohydrates. Interestingly, fructose and mannose metabolism were enriched in IFNβ-treated patients retaining NEDA status, whereas fatty acid degradation was enriched in CladT-treated patients. In our previous study comparing PwMS to healthy individuals, high intake of fructose, saturated fat, and trans fatty acids correlated with microbes enriched in PwMS [18]. These findings suggest that reducing the consumption of these nutrients could potentially facilitate the restoration of the gut microbiota—reversing the dysbiosis characteristic of PwMS—and, based on the results of the present study, may promote the attainment of NEDA status. The link between obesity or high-fat diets (generally rich in saturated fats) and MS risk, progression, and severity has been demonstrated in both EAE models and in PwMS, and both effective dietary interventions and microbiota-targeted treatments can be important supplemental strategies in MS management [91]. Low intake of iron, sodium, and sugar was associated in this study with IFNβ-induced IAE. We are not aware of any trial assessing the effects of specific diets on the severity of FLS in IFNβ-treated PwMS; however, patients are in general encouraged to follow a healthy diet to withstand FLS [92].

**Figure 5:**
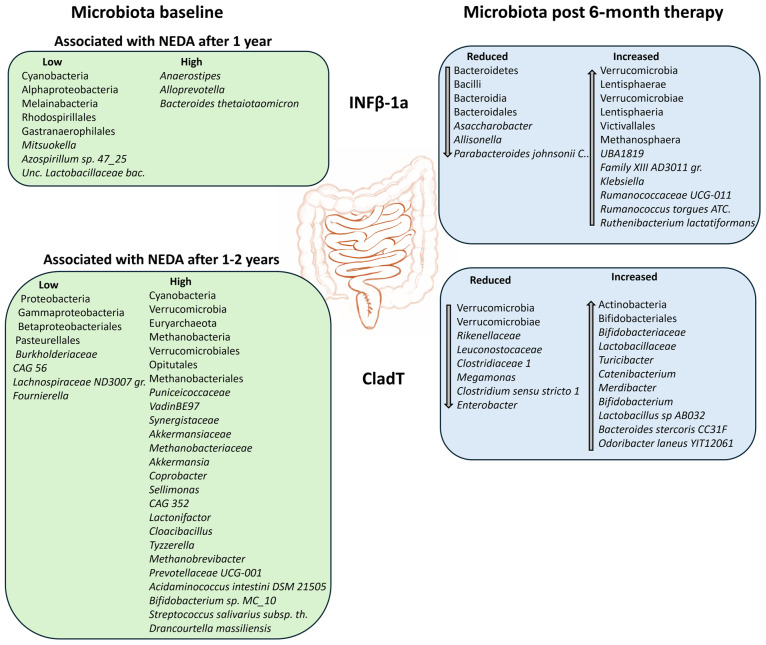
Summary of differentially abundant taxa for each DMT. (**Left**) Microbes at baseline significantly associated with NEDA status following 1 (IFNβ) or 1–2 years (CladT) of therapy. (**Right**) Microbes exhibiting significantly differential abundance after 6 months of therapy compared to baseline. Top: IFNβ-cohort; bottom: CladT cohort. Figure adopted from the National Institute of Diabetes and Digestive and Kidney Diseases, National Institutes of Health. Abbreviations: ATC.—ATCC277756, bac.—bacterium, C.—CL02T12C29, gr.—group, NEDA—no evidence of disease activity, th.—thermophilus.

A graphical summary of the results of this study is presented in Figure 5. In this longitudinal study, we investigated the impact of two MS-DMTs on the gut microbiota. One therapy belongs to the high-efficacy DMT class, and the other to the moderate-efficacy class, and accordingly, patient groups differed in disease duration and treatment history. Both treatments triggered beneficial shifts in the microbiota composition; however, no correlation was observed between therapeutic efficacy class and the magnitude of microbe alteration. While each DMT altered both overlapping and distinct taxa, we identified a consistent qualitative effect characterized by an increased abundance of SCFA-producing microbes, which were positively associated with favorable clinical outcomes. These accumulating results from various DMTs suggest that DMT-related modifications of the microbiota are both non-specific, potentially mediated through a general reduction in inflammation, and partially specific to their unique mode of action. Oral drugs may have a higher susceptibility to interact with gut microbes during metabolism [23], but interestingly, a study assessing iron administration found that iron modified the microbiota irrespective of the route of administration (intravenous, chronic transfusion or dietary supplementation) [93]. While DMT-induced increases in specific beneficial microbes may repair MS-associated dysbiosis towards a healthy microbiota, others may be part of a microbiota-mediated beneficial mechanism of action by which DMTs reduce the inflammatory state of the disease, promoting regulatory B and T cells and anti-inflammatory cytokines, and restoring intestinal integrity. In a large study by the iMSMS consortium, several taxa altered by DMTs did not differ in abundance between PwMS and healthy individuals [31], an observation consistent with our previous study on DMF-treated PwMS [28], suggesting that DMT-induced alterations do not exclusively restore a healthy microbiota. Microbiota-mediated changes in inflammation and disease activity in PwMS may include alterations in microbe-produced metabolites such as SCFAs, tryptophan/serotonin and bile acid derivatives, and microorganism-associated molecular patterns (MAMPs), all involved in immune cell function and activity [94]. An additional potential mechanism involves the modulation of intestinal IgA antibody responses by microbes or their metabolites. As the predominant secretory immunoglobulin in the gut, IgA is essential for preventing pathogen adhesion and translocation, clearance of bacterial toxins, and maintaining commensal microbiota homoeostasis. Furthermore, microbial shifts may contribute to repair the defective IgA-binding to gut microbiota observed in PwMS—a phenomenon that is more pronounced during active disease and correlates with disability [94,95,96,97,98,99]. Interestingly, a small cross-sectional study of CladT-treated PwMS revealed a non-significant trend toward increased serum IgA levels over time following treatment initiation [100].

A primary strength of this study is the longitudinal study design and the consistent technical conditions and homogeneous study populations under which IFNβ and CladT (as well as our previous DMF study [26]) were evaluated. This methodological consistency minimizes confounding factors and facilitates a comparison of how different DMTs modulate the microbiota. A primary limitation of this study is the relatively small cohort sizes, particularly regarding the number of samples available at the 6-month follow-up, which restricted the statistical power. Accordingly, further longitudinal validation studies of these DMTs are warranted. Moreover, the results of the DMT-induced compositional changes in the microbiota are primarily descriptive. Interpretations of these associations remain hypothetical, and larger-scale studies alongside functional experiments are required to establish causality and biological relevance. Another limitation is the taxonomic resolution limitations of 16S sequencing for microbial classification below the genus level.

In conclusion, our study suggests that microbiome modulation should be integrated into clinical decision making regarding therapeutic interventions for PwMS. Further investigation may reveal whether combining standard MS-DMT regimens with microbiome-targeted interventions, such as selected dietary modulations, pre-, pro- or post-biotics [75], or fecal microbiota transplant [45], can synergistically optimize the beneficial effects on the course of MS.

## 4. Material and Methods

### 4.1. Recruitment and Sample Collection

Persons with relapsing remitting MS about to initiate treatment with interferon beta-1a (Rebif) (*N* = 31) or CladT (Mavenclad) (*N* = 30) were recruited at the MS Center at Carmel Medical Center, Haifa, Israel, and all participants signed written informed consent. The study was conducted under a protocol approved by the Institutional Ethical Review Board of Carmel Medical Center (0019-20-CMC). Inclusion criteria included a diagnosis of relapsing remitting MS according to revised McDonald criteria [101], age 18–55 years, no intake of antibiotics/probiotics/corticosteroids within one month prior to recruitment, no other autoimmune disease, and no history of gastric/bowel surgery or irritable bowel disease (IBD). Fecal samples were collected using a stool preservative tube (Norgen Biotek, Thorold, ON, Canada) at recruitment and after 6 months of INFβ-1a or CladT therapy. Samples were frozen immidiately upon arrivel to the clinic and kept at −80° until DNA extraction. Demographic and clinical data was collected through a follow-up period of 1 year (INFβ-1a) and 2 years (CladT) from drug initiation. Data included level of disability (Expanded Disability Status Scale—EDSS), annual relapse rate calculated as mean over the last 2 years prior to recruitment, relapses since INFβ-1a/CladT initiation, and evidence of disease activity on MRI after ~1 year (both drugs) and 2 years (CladT) since drug initiation. Moreover, participants filled out a Mediterranean Diet Score (MDS) questionnaire [102,103,104] and a food frequency questionnaire (FFQ) [105,106,107] adapted to the Israeli population. The FFQs were analyzed at the Department of Public Health, Faculty of Health Sciences, Ben-Gurion University of the Negev, Beer-Sheva, Israel, for daily intake of energy and 78 various nutrients.

### 4.2. Clinical Response Definition

PwMS who discontinued INFβ-1a therapy within 1 year due to intolerable adverse events were defined as IAE, while patients that continued INFβ-1a therapy throughout follow-up were defined as patients without IAE. Clinical response was defined after 1 year (INFβ-1a) and after 1 and 2 years (CladT) of therapy as NEDA (No Evidence of Disease Activity) or Disease Activity (DA): clinical relapse, MRI activity (new or enlarged lesions or Gadolinium enhancement, compared to the previous MRI) and/or confirmed EDSS increase (for EDSS 0: ≥1.5 points; for EDSS 1.0–5.0: ≥1 point; for EDSS 5.5+: ≥0.5 point).

### 4.3. Microbial DNA Extraction

Microbial DNA sequencing was performed as previously described [18,28]. Briefly: DNA was extracted using the QIAamp^®^ PowerFecal^®^ Pro DNA kit (Qiagen, Tegelen, The Netherlands), according to manufacturer’s instructions, and the V3-V4 region of the 16s rRNA gene amplified using primers from the Earth Microbiome Project at Hy Laboratories Ltd. (Rehovot, Israel). Pooled samples were paired-end-sequenced using an Illumina Miseq v2 Kit (Illumina, Eindhoven, The Netherlands) at a depth of 100,000 reads/sample. After trimming reads for adaptor sequences and quality, reads were assigned to Operational Taxonomic Units (OTUs) for de novo picking against the SILVA database at >97% sequence similarity using the CLC-bio software version 12.0.3 (Qiagen, Tegelen, The Netherlands); 3933 and 3983 OTUs were identified in the INFβ-1a and CladT samples, respectively.

### 4.4. Statistical Analysis

Differences in MDS, EDSS and MSSS between pre- and post-treatment initiation were assessed by Wilcoxon’s paired test. Differences in MDS or specific nutrient intake between patients with NEDA status and patients with DA were assessed by the Mann–Whitney U test. Differences in nutrient intake between INFβ-1a-treated patients with and without IAE were assessed by independent t-test. Analyses was performed using IBM SPSS statistics version 28.0 (Armonk, NY, USA), and a *p*-value < 0.05 was considered significant. The microbiota data was analyzed using the MicrobiomeAnalyst web tool version 2.0 (Xia Lab, McGill University, Montreal, QC, Canada), with the Marker Data Profiling Module (MDP), according to established protocols [41,108,109]. OTUs were filtrated as ≥4 counts in at least 10% of samples. α-diversity (bacterial richness and evenness) was compared using the Shannon alpha index (Kruskal–Wallis test), and β-diversity (similarity and distance between samples) was compared using Bray–Curtis dissimilarity (PERMANOVA) on total sum scaling (TSS) normalized data. Statistical assessment of the differential relative abundance of microbes between 6-month post-treatment samples and baseline samples, between NEDA patients and patients with DA, or between patients with IAE and patients without IAE was performed using five different statistical packages within the MicrobiomeAnalyst web-tool, i.e., multiple linear regression (paired analysis), DESeq2 (normalization by relative log expression (RLE), EdgeR (normalization by trimmed mean of M-values (TMM), MetagenomeSeq (normalization by cumulative sum scaling (CSS). All were adjusted for multiple testing, at FDR < 0.1 (reflecting the exploratory nature of the study) as well as the Linear Discriminant Analysis Effective Size (LEfSe) tool (normalized by TSS) at a *p*-value < 0.05 and LDA ≥ ±1, following the recommendation to use multiple differential abundance methods for microbiota analysis [110]. Microbes exhibiting significantly differential abundance, as determined by at least two statistical methods, are presented in Section 2 (the results of all analyses are presented in the Supplementary Data). Comparing the microbe abundance of 6 months post CladT initiation versus baseline only, microbes exhibiting significantly differential abundance by at least one method at FDA ≤ 0.05 are presented. Correlations between the relative abundance of microbes identified as differential between patients maintaining NEDA versus DA for either IFNβ or CladT and ΔEDSS or ΔMSSS following 1 or 2 years of therapy were assessed by Spearman’s rank correlation, using IBM SPSS statistics (v28) at a *p*-value < 0.05 (abundance data normalized by TSS).

### 4.5. Functional Analysis

Differences in metabolic function, predicted from the gene content following 6 months of therapy versus baseline, or from baseline gene content in patients sustaining NEDA versus patients with DA after 1 or 2 years of therapy, were assessed using the Tax4Fun2 package within the MicrobiomeAnalyst web tool [40,109]. OTUs were filtered as described above, normalized by TSS, and then functions predicted using the KEGG orthologies (KOs). For enrichment analysis and metabolic network mapping, the KO abundance table was uploaded into the Shotgun Data Profiling module, filtered, and scaled by cumulative sum scaling (CSS). Enrichment of functional pathways following 6 months of treatment compared to baseline or between patients sustaining NEDA compared to patients with DA was assessed by Edge at FDR < 0.05 and by LEfSe at *p* < 0.05 and LDA > ±0.1.

## Figures and Tables

**Table 1:** Demographic and clinical data of participants.

Study Population	PwMS Pre-INFβ-1a *N* = 31	PwMS 6 Months INFβ-1a	PwMS 1 year INFβ-1a *n* = 29	PwMS Pre-CladT *N* = 30	PwMS 6 Months CladT	PwMS 1 Year CladT *n* = 27	PwMS 2 Years CladT *n* = 25
Age (y) mean ± SE	33.6 ± 1.7			36.8 ± 1.6			
Female (%)	87.1			76.7			
Ethnicity *n* (%) Jewish Arab	12 (39) 19 (61)			17 (57) 13 (43)			
Smoking (%)	16.1			43.3			
BMI (kg/m^2^)	25.1			23.1			
Vegetarian *n* (%)	1 (3.2)			2 (6.7)			
MDS mean ± SE [median] *p*-value (vs. pre-treatment)	6.5 ± 1.0 [6.0]	6.5 ± 0.6 [7.0] * *p* = 0.5		7.6 ± 0.3 [7.0]	7.3 ± 0.5 [6.0] * *p* = 0.3		
MDS *n* (%) Low (≤6) Intermediate (7–11) High ≥ 12 na	16 (51.6) 11 (35.6) 2 (6.5) 2 (6.5)	7 (41.2) 8 (47.1) 0 2 (11.8)		6 (20.0) 23 (76.7) 0 1 (3.3)	10 (53) 9 (47) 0 0		
Vitamin D (nmol/L) mean± SE [median]	54.5 ± 8 [52.6]			65.7 ± 7 [63.9]			
Disease duration (years)	0.87			8.9			
Previous DMT *n* (%) None (naïve) DMF Natalizumab Teriflunomide Fingolimod Interferon beta Siponimod DRF	24 (77.4) 4 (12.9) 1 (3.2) 1 (3.2) 1 (3.2)			4 (13.3) 11 (36.7) 5 (16.7) 3 (10) 2 (6.7) 3 (10) 2 (6.7)			
Time since previous DMT (months) mean ± SE [range]	3.9 ± 1.3 [1.0–9.1]			2.34 ± 0.1 [1.0–3.6]			
EDSS mean ± SE [median] *p*-value (vs. pre-treatment**)**	2.02 ± 0.47 [2]	1.56 ± 0.28 [1.0]	1.79 ± 0.53 [1.0] * *p* = 0.4	4.55 ± 0.3 [5.0]	4.32 ± 0.35 [5.0]	4.25 ± 0.36 [4.5] * *p* = 0.5	3.72 ± 0.5 [4.0] * *p* = 0.4
MSSS mean± SE *p*-value (vs. pre-treatment)	3.5 ± 0.9 [2.44]		3.6 ± 1.1 [2.01] * *p* = 0.5	5.6 ± 0.5 [5.82]		5.3 ± 0.6 [5.68] * *p* = 0.4	3.9 ± 0.6 [4.14] * *p* = 0.041
ARR prior to drug initiation mean ± SE	0.47 ± 0.1			0.7 ± 0.1			
Patients with NEDA *n* (%)			14 (48.3)			15 (50.0)	7 (23.3)
Patients with disease activity *n* (%)			10 (34.5)			12 (40.0)	18 (60)
Patients lost to follow-up *n*			2			3	5
Patients who discontinued treatment *n* (%)			5 (16)			1 (3.3)	6 (20)

Clinical and demographic data of PwMS pre-treatment, post 6 months, 1–2 years with IFNβ-1a or CladT therapy. * Statistical comparison by Wilcoxon’s paired test. Abbreviations: ARR—annual relapse rate, BMI—body mass index, DMF—dimethyl fumarate, DMT—disease-modifying therapy, DRF—diroximel fumarate, EDSS—Expanded Disability Status Scale, IAE—intolerable adverse events leading to drug discontinuation, MDS—Mediterranean diet score, MSSS—MS severity score, na—not available, NEDA—no evidence of disease activity, SE—standard of error.

**Table 2:** Differentially abundant microbes following 6 months of therapy. (**A**) INFβ-1a therapy versus baseline. (**B**) CladT therapy versus baseline.

(A)				(B)			
Microbes	FDR	FC (6 m vs. Pre)	Highest in	Microbes	FDR	FC (6 m vs. Pre)	Highest in
**Species**							
*Unc. Clostridiales bacterium*	0.020	4.87	6 m IFN	*Unc. Clostridiaceae bacterium*	^2^ 0.016		6 m CladT
*Parabacteroides johnsonii CL02T12C29*	0.020	0.22	Baseline	*Odoribacter laneus YIT 12061*	^2^ 0.003		6 m CladT
*Ruthenibacterium lactatiformans*	0.039	3.31	6 m IFN	*Unc. Lactobacillaceae bacterium*	^1^ 1.6 × 10^−13^	9 × 10^6^	6 m CladT
*Ruminococcus torques ATCC 27756*	0.006	6.23	6 m IFN	*Bacteroides stercoris CC31F*	^2^ 0.042		6 m CladT
*Unc. rumen bacterium*	0.003	5.31	6 m IFN	*Lactobacillus* sp. *AB032*	^2^ 0.042		6 m CladT
*Unc. archaeon*	0.006	10.2	6 m IFN				
**Genus**							
*Ruminococcaceae UCG-011*	2.6 × 10^−7^	7.42	6 m IFN	*Enterobacter*	^2^ 0.047		Baseline
*Klebsiella*	4.4 × 10^−6^	9.15	6 m IFN	*Clostridium sensu stricto 1*	^3^ 0.019	LDA −3.5	Baseline
*Family XIII AD3011 group*	0.020	2.66	6 m IFN	*Megamonas*	0.0041	0.04	Baseline
*Allisonella*	0.075	0.24	Baseline	*Bifidobacterium*	^3^ 0.036	LDA 4.2	6 m CladT
*UBA1819*	0.075	2.84	6 m IFN	*Merdibacter*	^3^ 0.015	LDA 1.4	6 m CladT
*Methanosphaera*	0.031	6.66	6 m IFN	*Catenibacterium*	^2^ 0.047		6 m CladT
*Asaccharobacter*	0.092	0.39	Baseline	*Turicibacter*	^3^ 0.041	LDA 1.8	6 m CladT
**Family**							
*Gut metagenome*	0.054	3.44	6 m IFN	*Clostridiaceae 1*	^3^ 0.021	LDA−3.5	Baseline
				*Lactobacillaceae*	^1^ 0.002	12.5	6 m CladT
				*Bifidobacteriaceae*	^3^ 0.036	LDA 4.2	6 m CladT
				*Leuconostocaceae*	^2^ 0.05		Baseline
				*Rikenellaceae*	^2^ 0.05		Baseline
**Order**							
Bacteroidales	0.095	0.54	Baseline	Bifidobacteriales	^3^ 0.036	LDA 4.2	6 m CladT
Victivallales	0.032	3.99	6 m IFN				
**Class**							
Bacteroidia	^3^ 0.049	LDA −4.4	Baseline	Actinobacteria	^3^ 0.036	LDA 4.2	6 m CladT
Verrucomicrobiae	0.002	7.07	6 m IFN	Verrucomicrobiae	0.05	0.23	Baseline
Bacilli	0.030	0.19	Baseline				
Lentisphaeria	0.032	3.62	6 m IFN				
**Phylum**							
Verrucomicrobia	0.0006	6.85	6 m IFN	Verrucomicrobia	0.04	0.25	Baseline
Bacteroidetes	0.035	0.46	Baseline				
Lentisphaerae	0.006	3.74	6 m IFN				

Differentially abundant microbes following 6 months of (A)—INFβ-1a or (B)—CladT therapy compared to baseline. Representative significant statistical method: EdgeR or ^1^ DeSeq2, ^2^ MetagenomeSeq, ^3^ LEfSe. For INFβ-1a, differential microbes significant by at least two statistical methods at FDA < 0.1 are presented. For CladT, differential microbes significant by at least one method at FDA ≤ 0.05 are presented. For ^3^ LEfSe, *p*-value < 0.05 is presented. Abbreviations: FC—fold change, FDR—false discovery rate, IFN—INFβ-1a, LDA—linear discriminant analysis, LEfSe—LDA effect size, m—months, Pre—pre-treatment, unc.—uncultured, vs.—versus.

**Table 3:** Differential microbial abundance in INFβ-1a-treated patients remaining NEDA versus patients exhibiting disease activity after 1 year of therapy. (**A**) Samples obtained at baseline. (**B**) Samples obtained following 6 months of therapy.

(A)				(B)			
Microbes	FDR	FC	Highest in	Microbes	FDR	FC	Highest in
**Species**							
*Azospirillum* sp. *47_25*	0.005	0.1	DA				
*Bacteroides* *thetaiotaomicron*	0.033	10.9	NEDA				
*Unc. Lactobacillaceae* *bacterium*	0.078	0.19	DA				
**Genus**							
*Mitsuokella*	1 × 10^−5^	0.01	DA	*Enterococcus*	0.024	0.09	DA
*Alloprevotella*	0.0002	175	NEDA	*Klebsiella*	0.067	0.08	DA
*Anaerostipes*	0.036	5.5	NEDA				
**Family**							
				*Enterococcaceae*	0.009	0.08	DA
**Order**							
Gastranaerophilales	0.0002	0.05	DA	Aeromonadales	2 × 10^−8^	0.003	DA
Rhodospirillales	0.026	0.11	DA				
**Class**							
Melainabacteria	0.001	0.08	DA				
Alphaproteobacteria	0.029	0.13	DA				
**Phylum**							
Cyanobacteria	3 × 10^−5^	0.05	DA				

Microbes exhibiting differential relative abundance between patients remaining NEDA and those with disease activity after 1 year of therapy. Data are shown for (A) baseline samples (*n* = 24) and (B) samples obtained following 6 months of INFβ-1a therapy (*n* = 17). Only results significant by at least two statistical methods are presented (FDR from EdgeR shown). Abbreviations: DA—disease activity, FC—fold change, FDR—false discovery rate, NEDA—no evidence of disease activity, unc.—uncultured.

**Table 4:** Differential microbe abundance in CladT-treated patients remaining NEDA versus patients exhibiting disease activity after 1 year of therapy. (**A**) Samples obtained at baseline. (**B**) Samples obtained following 6 months of therapy.

(A)				(B)			
Microbes	FDR	FC	Highest in	Microbes	FDR	FC	Highest in
**Species**							
*Acidaminococcus intestine DSM 21505*	0.033	8.74	NEDA	*Massiliprevotella massiliensis*	0.063	10.2	NEDA
*Bifidobacterium* sp. *MC 10*	0.042	6.1	NEDA	*Lactobacillus* sp. *AB032*	^1^ 0.059	37.1	NEDA
*Unc. Ruminococcus* sp.	0.058	16.4	NEDA	*Bacteroides ovatus V975*	0.005	LDA−2.7	DA
				*Azospirillum* sp. *47_25*	0.003	0.03	DA
				*Streptococcus salivarius subsp thermophilus*	0.085	13.1	NEDA
				*Bacteroides eggerthii DSM 20697*	0.086	15.8	NEDA
				*Unc. archaeon*	0.059	17.6	NEDA
**Genus**							
*CAG-56*	0.039	0.12	DA	*Azospirillum* sp. *47_25*	7 × 10^−5^	0.03	DA
*Methanobrevibacter*	^1^ 0.016	LDA 3.0	NEDA	*Methanobrevibacter*	0.002	87.9	NEDA
*Tyzzerella 3*	0.052	7.0	NEDA	*Parasutterella*	0.021	0.15	DA
*Cloacibacillus*	0.067	5.0	NEDA	*Rikenellaceae RC9 gut group*	0.021	41.2	NEDA
*Coprobacter*	0.095	3.48	NEDA	*Desulfovibrio*	0.055	10.6	NEDA
*Akkermansia*	0.042	10.5	NEDA	*Prevotella 7*	0.055	12.8	NEDA
*Prevotellaceae UCG 001*	0.039	11.0	NEDA	*Eisenbergiella*	0.055	8.54	NEDA
				*Phascolarctobacterium*	0.060	9.62	NEDA
				*Catenibacterium*	0.067	25.4	NEDA
				*Mogibacterium*	0.087	9.87	NEDA
				*Clostridium sensu stricto 1*	^1^ 0.014	LDA 3.2	NEDA
**Family**							
*Methanobacteriaceae*	^1^ 0.016	LDA 3.1	NEDA	*Methanobacteriaceae*	0.001	60.6	NEDA
*Akkermansiaceae*	0.040	7.0	NEDA				
*Synergistaceae*	0.045	4.7	NEDA				
*VadinBE97*	0.074	3.0	NEDA				
*Puniceicoccaceae*	^1^ 0.034	LDA 2.1	NEDA				
*Burkholderiaceae*	0.040	0.26	DA				
**Order**							
Methanobacteriales	^1^ 0.016	LDA 3.1	NEDA	Methanobacteriales	0.002	50.4	NEDA
Pasteurellales	0.028	0.22	DA	Synergistales	0.035	11.2	NEDA
Betaproteobacteriales	0.028	0.26	DA	Betaproteobacteriales	^1^ 0.028	LDA−3.1	DA
Opitutales	^1^ 0.034	LDA 2.1	NEDA				
Verrucomicrobiales	0.076	5.35	NEDA				
**Class**							
Methanobacteria	^1^ 0.016	LDA 3.1	NEDA	Methanobacteria	0.0014	49.8	NEDA
**Phylum**							
Euryarchaeota	0.023	5.41	NEDA	Euryarchaeota	0.001	36.2	NEDA
Verrucomicrobia	0.0006	9.0	NEDA				

Microbes exhibiting differential relative abundance between patients remaining NEDA and those with disease activity after 1 year of therapy. Data are shown for (A) baseline samples (*n* = 27) and (B) samples obtained following 6 months of CladT therapy (*n* = 19). Only results significant by at least two statistical methods are presented (FDR from EdgeR shown, ^1^ *p*-value from LEfSe). Abbreviations: DA—disease activity, FC—fold change, FDR—false discovery rate, LDA—linear discriminant analysis, LEfSe—LDA effect size, NEDA—no evidence of disease activity, unc.—uncultured.

**Table 5:** Differential microbe abundance in CladT-treated patients remaining NEDA versus patients exhibiting disease activity after 2 years of therapy. (**A**) Samples obtained at baseline. (**B**) Samples obtained following 6 months of therapy.

(A)				(B)			
Microbes	FDR	FC	Highest in	Microbes	FDR	FC	Highest in
**Species**							
*Drancourtella* *massiliensis*	0.0002	22.2	NEDA	*Ruthenibacterium lactatiformans*	0.0006	9.02	NEDA
*Bifidobacterium* sp. *MC 10*	0.0011	8.54	NEDA	*Lactobacillus* sp. *AB032*	5.9 × 10^−6^	81.3	NEDA
*Acidaminococcus intestine DSM 21505*	0.0013	8.95	NEDA	*Acidaminococcus intestine DSM 21505*	0.014	11.6	NEDA
*Streptococcus salivarius subsp. thermophilus*	0.0013	12.0	NEDA	*Unc. Methanobrevibacter sp*	^1^ 0.034	LDA 1.39	NEDA
				*Unc. archaeon*	1.8 × 10^−7^	33.7	NEDA
**Genus**							
*CAG-352*	0.001	47.9	NEDA	*Tyzzerella 4*	3.6 × 10^−6^	147	NEDA
*Sellimonas*	0.0031	14.8	NEDA	*Methanosphaera*	8.3 × 10^−6^	23.8	NEDA
*Lactonifactor*	0.0054	5.14	NEDA	*Synergistes*	0.00001	40.4	NEDA
*Fournierella*	0.045	0.09	DA	*Eubacterium*	0.00008	14.1	NEDA
*Lachnospiraceae ND3007 group*	^1^ 0.04	LDA −3.3	DA	*Eisenbergiella*	0.0003	11.2	NEDA
				*Coprobacillus*	0.0005	10.6	NEDA
				*Methanobrevibacter*	0.001	18.3	NEDA
				*Tyzzerella 3*	0.019	0.03	DA
				*UBA1819*	0.0097	6.8	NEDA
				*Libanicoccus*	0.0083	11.7	NEDA
**Family**							
				*Synergistaceae*	0.0001	21.0	NEDA
				*Eubacteriaceae*	0.002	9.55	NEDA
				*Muribaculaceae*	0.0034	7.4	NEDA
				*Methanobacteriaceae*	0.0026	13.5	NEDA
				*Puniceicoccaceae*	0.027	4.61	NEDA
**Order**							
Betaproteobacteriales	^1^ 0.025	LDA −3.1	DA	Synergistales	0.0001	18.0	NEDA
Opitutales	^1^ 0.024	LDA 1.8	NEDA	Opitutales	^1^ 0.026	LDA 2.4	NEDA
Methanobacteriales	^1^ 0.018	LDA 2.1	NEDA	Methanobacteriales	0.0012	12.5	NEDA
**Class**							
Gammaproteobacteria	0.047	0.18	DA	Synergistia	0.00005	20.9	NEDA
				Methanobacteria	0.0004	13.5	NEDA
				Mollicutes	0.099	0.1	DA
**Phylum**							
Euryarchaeota	0.014	6.13	NEDA	Euryarchaeota	0.0002	15.9	NEDA
Cyanobacteria	^1^ 0.018	LDA 2.1	NEDA	Synergistetes	9.6 × 10^−6^	23.4	NEDA
Proteobacteria	0.014	0.18	DA	Tenericutes	0.088	0.13	DA

Microbes exhibiting differential relative abundance between patients remaining NEDA and those with disease activity after 2 years of therapy. Data are shown for (A) baseline samples (*n* = 27) and (B) samples obtained following 6 months of CladT therapy (*n* = 19). Only results significant by at least two statistical methods are presented (FDR from EdgeR shown, ^1^ *p*-value from LEfSe). Abbreviations: DA—disease activity, FC—fold change, FDR—false discovery rate, LDA—linear discriminant analysis, LEfSe—LDA effect size, NEDA—no evidence of disease activity, unc.—uncultured.

**Table 6:** (**A**) NEDA- or DA-associated microbes correlating with ΔEDSS or ΔMSSS after 1 year of IFNβ therapy. (**B**) NEDA- or DA-associated microbes correlating with ΔEDSS or ΔMSSS after 1 or 2 years of CladT therapy.

**(A)**																
	**Baseline Samples**								**6-Month Samples**							
**Microbes**	Δ**EDSS 1Y**				Δ**MSSS 1Y**				Δ**EDSS 1Y**				Δ**MSSS 1Y**			
	***p*** **-Value**		**r**		***p*** **-Value**		**r**		***p*** **-Value**		**r**		***p*** **-Value**		**r**	
Alphaproteobacteria	**0.033**		0.44		**0.001**		0.73									
Anaerostipes	0.061		−0.39		**0.023**		−0.53									
Bacteroides thetaiotaomicron									0.090		−0.47					
Enterococcaceae	**0.029**		0.45													
Enterococcus	**0.029**		0.45													
Mitsuokella	**0.034**		0.42						**0.015**		0.63					
Rhodospirillales	**0.033**		0.44		**0.001**		0.73		0.094		0.47		0.067		0.50	
**(B)**																
	**Baseline Samples**								**6-Month Samples**							
	Δ**EDSS 1Y**		Δ**MSSS 1Y**		Δ**EDSS 2Y**		Δ**MSSS 2Y**		Δ**EDSS 1Y**		Δ**MSSS 1Y**		Δ**EDSS 2Y**		Δ**MSSS 2Y**	
**Microbes**	***p*** **-Value**	**r**	***p*** **-Value**	**r**	***p*** **-Value**	**r**	***p*** **-Value**	**r**	***p*** **-Value**	**r**	***p*** **-Value**	**r**	***p*** **-Value**	**r**	***p*** **-Value**	**r**
Euryarchaeota	**0.008**	−0.52	**0.023**	−0.49	0.093	−0.40										
Methanobacteria	**0.008**	−0.52	**0.023**	−0.49	0.093	−0.40										
Methanobacteriales	**0.008**	−0.52	**0.023**	−0.49	0.093	−0.40										
Methanobacteriaceae	**0.008**	−0.52	**0.023**	−0.49	0.093	−0.40										
Methanobrevibacter	**0.006**	−0.54	**0.02**	−0.50	0.069	−0.43										
Lactonifactor	**0.041**	−0.41	**0.03**	−0.47					**0.015**	−0.56	**0.028**	−0.58				
Pasteurellales	0.055	0.39														
Verrucomicrobia	**0.036**	−0.42														
Opitutales	**0.049**	−0.40														
Puniceicoccaceae	**0.049**	−0.40														
Acidaminococcus intestini DSM 21505			**0.025**	−0.49					**0.02**	−0.54	**0.014**	−0.64				
CAG-352							0.085	−0.46								
Lachnospiraceae ND3007 group									**0.024**	0.53	**0.043**	0.55				
Tyzzerella 3											**0.046**	0.54				
Coprobacter													**0.022**	0.60	**0.030**	0.68

Spearman’s rank correlations were calculated between relative abundance (at baseline or after 6 months of therapy) and clinical progression (ΔEDSS or ΔMSSS). (A) IFNβ cohort: microbes were selected based on associations with NEDA or DA identified in Table 4 and assessed at the 1-year follow-up. (B) CladT cohort: microbes were selected based on associations with NEDA or DA identified in Table 5 and Table 6 and assessed at the 1-and 2-year follow-ups. Significant correlations are highlighted in bold. Abbreviation: DA—disease activity, EDSS—Expanded Disability Status Scale, MSSS—Multiple Sclerosis Severity Score, NEDA—no evidence of disease activity, r—correlation coefficient, Y-year.

**Table 7:** Predicted functional pathways enriched in (**A**) the IFNβ cohort and (**B**) the CladT cohort.

(A)					(B)				
Pathway	Total	Expected	Hits	*p* Value	Pathway	Total	Expected	Hits	*p* Value
1—Enriched pathways: 6 months post-therapy versus baseline									
Glycosaminoglycan degradation	13	0.02	1	0.020	Propanoate metabolism	82	0.33	4	0.0002
Drug metabolism—other enzymes	20	0.03	1	0.031	Galactose metabolism	58	0.23	2	0.022
* Sulfur metabolism	79	0.52	3	0.014	Other glycan degradation	9	0.04	1	0.036
* Biosynthesis of siderophore group nonribosomal peptides	4	0.03	1	0.026					
* Sesquiterpenoid and triterpenoid biosynthesis	5	0.03	1	0.032					
* Ubiquinone and other terpenoid-quinone biosynthesis	46	0.3	2	0.035					
* Citrate cycle (TCA cycle)	54	0.35	2	0.047					
* Valine, leucine and isoleucine degradation	55	0.36	2	0.049					
2—Enriched pathways: NEDA versus DA (1 year)									
Fructose and mannose metabolism	94	1.43	9	8 × 10^−6^	Valine, leucine and isoleucine degradation	55	0.58	5	0.0002
Fatty acid degradation	25	0.38	5	3 × 10^−5^	Butanoate metabolism	97	1.02	6	0.0004
Butanoate metabolism	97	1.48	8	8 × 10^−5^	Fatty acid degradation	25	0.26	3	0.002
Valine, leucine and isoleucine degradation	55	1.56	6	0.0002	Phenylalanine, tyrosine and tryptophan biosynthesis	59	0.62	3	0.023
Lysine degradation	46	0.7	5	0.0006	Lipoic acid metabolism	25	0.26	2	0.028
Inositol phosphate metabolism	32	0.49	4	0.001	Methane metabolism	173	1.83	5	0.033
Benzoate degradation	86	1.31	6	0.002	beta-Alanine metabolism	32	0.34	2	0.044
Pinene, camphor and geraniol degradation	7	0.11	2	0.005	Propanoate metabolism	82	0.87	3	0.054
Ascorbate and aldarate metabolism	46	0.7	4	0.005	3—Enriched pathways: NEDA versus DA (2 years)				
Galactose metabolism	58	0.88	4	0.011	Methane metabolism	173	1.02	10	9 × 10^−9^
beta-Alanine metabolism	32	0.49	3	0.012	Pantothenate and CoA biosynthesis	38	0.22	2	0.020
Aminobenzoate degradation	33	0.50	3	0.013					
Propanoate metabolism	82	1.25	4	0.035					

Enriched pathways were mapped according to predicted functional profiles (KOs) in the (A) IFNβ or (B) CladT cohort. (1) Enriched pathways at 6 months post-therapy versus baseline (LEfSe, *p* < 0.05, LDA > ±0.1 or * Edge, FDR < 0.05). (2) Enriched pathways in NEDA patients versus those with DA after 1 year of therapy (LEfSe, *p* < 0.05, LDA > ±0.1). (3) Enriched pathways in NEDA patients versus those with DA after 2 years of therapy (LEfSe, *p* < 0.05, LDA > ±0.05). Abbreviations: DA—disease activity, KO—Encyclopedia of Genes and Genomes orthologs (KEGG) orthology; NEDA—no evidence of disease activity.

## Data Availability

The original contributions presented in this study are included in the article/Supplementary Material. Further inquiries can be directed to the corresponding author.

## Supplementary Materials

The following supporting information can be downloaded at: https://www.mdpi.com/article/10.3390/ijms27083500/s1.

## References

[1] Freedman S.N., Shahi S.K., Mangalam A.K. (2018). The “Gut Feeling”: Breaking Down the Role of Gut Microbiome in Multiple Sclerosis. Neurotherapeutics.

[2] Valdes A., Walter J., Segal E., Spector T.D. (2018). Role of the gut microbiota in nutrition and health. BMJ.

[3] Krishnan S., Alden N., Lee K. (2015). Pathways and Functions of Gut Microbiota Metabolism Impacting Host Physiology. Curr. Opin. Biotechnol..

[4] Rinninella E., Raoul P., Cintoni M., Franceschi F., Abele G., Miggiano D., Gasbarrini A., Mele M.C. (2019). What is the healthy gut microbiota composition ? A changing ecosystem across age, environment, diet, and diseases. Microorganisms.

[5] Elsayed N.S., Aston P., Bayanagari V.R., Shukla S.K. (2022). The gut microbiome molecular mimicry piece in the multiple sclerosis puzzle. Front. Immunol..

[6] Del Negro I., Pez S., Varsace S., Marziali A., Gigli G., Tereshko Y., Valente M. (2024). Impact of Disease-Modifying Therapies on Gut—Brain Axis in Multiple Sclerosis. Medicina.

[7] Loh J.S., Mak W.Q., Kar L., Tan S., Ng C.X., Chan H.H., Yeow S.H., Foo J.B., Ong Y.S. (2024). Microbiota-Gut-Brain axis and its therapeutic applications in neurodegenerative diseases. Signal Transduct. Target. Ther..

[8] Parodi B., De Rosbo N.K. (2021). The Gut-Brain Axis in Multiple Sclerosis. Is Its Dysfunction a Pathological Trigger or a Consequence of the Disease?. Front. Immunol..

[9] Jangi S., Gandhi R., Cox L.M., Li N., von Glehn F., Yan R., Patel B., Mazzola M.A., Liu S., Glanz B.L. (2016). Alterations of the human gut microbiome in multiple sclerosis. Nat. Commun..

[10] Ventura R.E., Iizumi T., Battaglia T., Liu M., Perez-Perez G.I., Herbert J., Blaser M.J. (2019). Gut microbiome of treatment-naïve MS patients of different ethnicities early in disease course. Sci. Rep..

[11] Chen J., Chia N., Kalari K.R., Yao J.Z., Novotna M., Soldan M.M.P., Luckey D.H., Marietta E.V., Jeraldo P.R., Chen X. (2016). Multiple sclerosis patients have a distinct gut microbiota compared to healthy controls. Sci. Rep..

[12] Correale J., Hohlfeld R., Baranzini S.E. (2022). The role of the gut microbiota in multiple sclerosis. Nat. Rev. Neurol..

[13] Galluzzo P., Capri F.C., Vecchioni L., Realmuto S., Scalisi L., Cottone S., Nuzzo D., Alduina R. (2021). Comparison of the intestinal microbiome of italian patients with multiple sclerosis and their household relatives. Life.

[14] Mielcarz D.W., Kasper L.H. (2015). The Gut Microbiome in Multiple Sclerosis. Curr. Treat. Options Neurol..

[15] Mirza A., Forbes J.D., Zhu F., Bernstein C.N., Van Domselaar G., Graham M., Waubant E., Tremlett H. (2020). The multiple sclerosis gut microbiota: A systematic review. Mult. Scler. Relat. Disord..

[16] Noto D., Miyake S. (2022). Gut dysbiosis and multiple sclerosis. Clin. Immunol..

[17] Ordoñez-Rodriguez A., Roman P., Rueda-Ruzafa L., Campos-Rios A., Cardona D. (2023). Changes in gut microbiota and Multiple Sclerosis: A systematic review. Int. J. Environ. Res. Public Health.

[18] Nitzan Z., Staun-Ram E., Volkowich A., Miller A. (2023). Multiple Sclerosis-Associated Gut Microbiome in the Israeli Diverse Populations: Associations with Ethnicity, Gender, Disability Status, Vitamin D Levels, and Mediterranean Diet. Int. J. Mol. Sci..

[19] Montgomery T.L., Wang Q., Mirza A., Dwyer D., Wu Q., Dowling C.A., Martens J.W.S., Yang J., Krementsov D.N. (2024). Identification of commensal gut microbiota signatures as predictors of clinical severity and disease progression in multiple sclerosis. Sci. Rep..

[20] Schwerdtfeger L.A., Montini F., Lanser T.B., Chitnis T., Cox L.M., Weiner H.L., Schwerdtfeger L.A., Montini F., Lanser T.B., Ekwudo M.N. (2025). Gut microbiota and metabolites are linked to disease progression in multiple sclerosis. Cell Rep. Med..

[21] Tremlett H., Fadrosh D.W., Faruqi A.A., Hart J., Roalstad S., Graves J., Lynch S., Waubant E., Aaen G., Belman A. (2016). Gut microbiota composition and relapse risk in pediatric MS: A pilot study. J. Neurol. Sci..

[22] Horton M.K., Mccauley K., Fadrosh D., Fujimura K., Graves J., Ness J., Wheeler Y., Gorman M.P., Benson L.A., Weinstock-guttman B. (2021). Gut microbiome is associated with multiple sclerosis activity in children. Ann. Clin. Transl. Neurol..

[23] Steiner H.E., Patterson H.K., Giles J.B. (2022). Bringing pharmacomicrobiomics to the clinic through well-designed studies. Clin. Transl. Sci..

[24] Vacaras V., Muresanu D.F., Buzoianu A., Nistor C., Vesa C., Paraschiv A., Botos-vacaras D., Vacaras C., Vithoulkas G. (2023). The role of multiple sclerosis therapies on the dynamic of human gut microbiota. J. Neuroimmunol..

[25] Tsai C., Jette S., Tremlett H. (2023). Disease-modifying therapies used to treat multiple sclerosis and the gut microbiome: A systematic review. J. Neurol..

[26] Cantarel B.L., Waubant E., Chehoud C., Kuczynski J., DeSantis T.Z., Warrington J., Venkatesan A., Fraser C.M., Mowry E.M. (2015). Gut microbiota in multiple sclerosis: Possible influence of immunomodulators. J. Investig. Med..

[27] Katz Sand I., Zhu Y., Ntranos A., Clemente J.C., Cekanaviciute E., Brandstadter R., Crabtree-Hartman E., Singh S., Bencosme Y., Debelius J. (2019). Disease-modifying therapies alter gut microbial composition in MS. Neurol. Neuroimmunol. Neuroinflamm..

[28] Staun-ram E., Volkowich A., Miller A. (2025). Immunotherapy-mediated modulation of the gut microbiota in multiple sclerosis and associations with diet and clinical response—The effect of dimethyl fumarate therapy. Ther. Adv. Neurol. Disord..

[29] Storm-Larsen C., Myhr K., Farbu E., Midgard R., Nyquist K., Broch L., Buness A., Holm K., Ueland T., Fallang L. (2019). Gut microbiota composition during a 12-week intervention with delayed-release dimethyl fumarate in multiple sclerosis—A pilot trial. Mult. Scler. J. Exp. Transl. Clin..

[30] Diebold M., Meola M., Purushothaman S., Siewert L.K. (2022). Gut microbiota composition as a candidate risk factor for dimethyl fumarate-induced lymphopenia in multiple sclerosis. Gut Microbes.

[31] Zhou X., Baumann R., Gao X., Mendoza M., Singh S., Katz Sand I., Xia Z., Cox L.M., Chitnis T., Yoon H. (2022). Gut microbiome of multiple sclerosis patients and paired household healthy controls reveal associations with disease risk and course. Cell.

[32] Ferri C., Castellazzi M., Merli N., Laudisi M., Baldin E., Baldi E., Mancabelli L., Ventura M., Pugliatti M. (2023). Gut Microbiota Changes during Dimethyl Fumarate Treatment in Patients with Multiple Sclerosis. Int. J. Mol. Sci..

[33] Selmaj K., Cree B.A.C., Barnett M., Thompson A., Peter H. (2024). Multiple sclerosis: Time for early treatment with high—Efficacy drugs. J. Neurol..

[34] Edan G., Le E. (2023). Escalation Versus Induction/High—Efficacy Treatment Strategies for Relapsing Multiple Sclerosis: Which is Best for Patients?. Drugs.

[35] Giles E.M., Stagg A.J. (2017). Type 1 Interferon in the Human Intestine—A Co-ordinator of the Immune Response to the Microbiota. Inflamm. Bowel Dis..

[36] Kozhieva M., Naumova N., Alikina T., Boyko A., Vlassov V., Kabilov M.R. (2021). The Core of Gut Life: Firmicutes Profile in Patients with Relapsing-Remitting Multiple Sclerosis. Life.

[37] Castillo-Álvarez F., Pérez-Matute P., Oteo J.A.A., Marzo-Sola M.E.E. (2021). The influence of interferon β-1b on gut microbiota composition in patients with multiple sclerosis. Neurología.

[38] Paraschiv A., Vacaras V., Nistor C., Vacaras C., Strilciuc S. (2024). The effect of multiple sclerosis therapy on gut microbiota dysbiosis: A longitudinal prospective study. Microb. Cell.

[39] van Pamelen J., Rodriguez-Mogeda C., van Olst L., van der Pol S.M.A., Boon M.L., De Beukelaar J., Gerlach O.H.H., Budding A.E., Killestein J., De Vries H.E. (2025). The gut-brain-axis one year after treatment with cladribine tablets in patients with relapsing remitting multiple sclerosis: A pilot study. Front. Immunol..

[40] Wemheuer F., Taylor J.A., Daniel R., Johnston E., Meinicke P., Thomas T., Wemheuer B. (2020). Tax4Fun2: Prediction of habitat-specific functional profiles and functional redundancy based on 16S rRNA gene sequences. Environ. Microbiomes.

[41] Chong J., Liu P., Zhou G., Xia J. (2020). Using MicrobiomeAnalyst for comprehensive statistical, functional, and meta-analysis of microbiome data. Nat. Protoc..

[42] Berer K., Mues M., Koutrolos M., AlRasbi Z., Boziki M., Johner C., Wekerle H., Krishnamoorthy G., Rasbi Z.A., Boziki M. (2011). Commensal microbiota and myelin autoantigen cooperate to trigger autoimmune demyelination—With comments. Nature.

[43] Berer K., Gerdes L.A., Cekanaviciute E., Jia X., Xiao L., Xia Z., Liu C., Klotz L., Stauffer U., Baranzini S.E. (2017). Gut microbiota from multiple sclerosis patients enables spontaneous autoimmune encephalomyelitis in mice. Proc. Natl. Acad. Sci. USA.

[44] Cekanaviciute E., Yoo B.B., Runia T.F., Debelius J.W., Singh S., Nelson C.A., Kanner R., Bencosme Y., Lee Y.K., Hauser S.L. (2017). Gut bacteria from multiple sclerosis patients modulate human T cells and exacerbate symptoms in mouse models. Proc. Natl. Acad. Sci. USA.

[45] Laeeq T., Vongsavath T., Tun K.M. (2023). The Potential Role of Fecal Microbiota Transplant in the Reversal or Stabilization of Multiple Sclerosis Symptoms: A Literature Review on Efficacy and Safety. Microorganisms.

[46] Jiang J., Chu C., Wu C., Wang C., Zhang C., Li T., Zhai Q., Yu L., Tian F., Chen W. (2021). Efficacy of probiotics in multiple sclerosis: A systematic review of preclinical trials and meta-analysis of randomized controlled trials. Food Funct..

[47] Asghari K.M., Dolatkhah N., Ayromlou H., Mirnasiri F., Dadfar T., Hashemian M. (2023). The effect of probiotic supplementation on the clinical and para-clinical findings of multiple sclerosis: A randomized clinical trial. Sci. Rep..

[48] Wilkinson E.M., Ilhan Z.E., Herbst-Kralovetz M.M. (2018). Microbiota–drug interactions: Impact on metabolism and efficacy of therapeutics. Maturitas.

[49] Ganamurali N., Sabarathinam S. (2025). Microbial modulation of digoxin bioavailability: A pharmacomicrobiome perspective on Eggerthella lenta’s role in steroid-like drug metabolism and precision therapeutics. J. Steroid Biochem. Mol. Biol..

[50] Haiser H.J., Gootenberg D.B., Chatman K., Sirasani G., Balskus E.P., Turnbaugh P.J. (2013). Predicting and manipulating cardiac drug inactivation by the human gut bacterium *Eggerthella lenta*. Science.

[51] Troci A., Zimmermann O., Esser D., Krampitz P., May S., Franke A., Berg D., Leypoldt F., Stürner K.H., Bang C. (2022). B-cell-depletion reverses dysbiosis of the microbiome in multiple sclerosis patients. Sci. Rep..

[52] Cox L.M., Maghzi A.H., Liu S., Tankou S.K., Dhang F.H., Willocq V., Song A., Wasén C., Tauhid S., Chu R. (2021). Gut Microbiome in Progressive Multiple Sclerosis. Ann. Neurol..

[53] Tremlett H., Zhu F., Arnold D., Bar-Or A., Bernstein C.N., Bonner C., Forbes J.D., Graham M., Hart J., Knox N.C. (2021). The gut microbiota in pediatric multiple sclerosis and demyelinating syndromes. Ann. Clin. Transl. Neurol..

[54] Bui T.N.Y., Paul A., Guleria S., Sullivan J.M.O., Toldi G. (2025). Short-chain fatty acids—A key link between the gut microbiome and T-lymphocytes in neonates?. Pediatr. Res..

[55] Kujawa D., Laczmanski L., Budrewicz S., Pokryszko- A., Podbielska M. (2023). Targeting gut microbiota: New therapeutic opportunities in multiple sclerosis. Gut Microbes.

[56] Sanchez J.M.S., DePaula-Silva A.B., Libbey J.E., Fujinami R.S. (2022). Role of diet in regulating the gut microbiota and multiple sclerosis. Clin. Immunol..

[57] Haghikia A., Jörg S., Duscha A., Berg J., Manzel A., Waschbisch A., Hammer A., Lee D.-H., May C., Wilck N. (2015). Dietary fatty acids directly impact Central Nervous System autoimmunity via the small intestine. Immunity.

[58] Golpour F., Abbasi-alaei M., Babaei F., Mirzababaei M., Parvardeh S., Mohammadi G., Nassiri-asl M. (2023). Biomedicine & Pharmacotherapy Short chain fatty acids, a possible treatment option for autoimmune diseases. Biomed. Pharmacother..

[59] Freedman M.S., Coyle P.K., Hellwig K., Singer B., Wynn D., Andrew S.M., Fernando G., Korich J., Reder A.T. (2024). Twenty Years of Subcutaneous Interferon-Beta-1a for Multiple Sclerosis: Contemporary Perspectives. Neurol. Ther..

[60] Wirusanti N.I., Baldridge M.T., Harris V.C. (2022). Microbiota regulation of viral infections through interferon signaling. Trends Microbiol..

[61] Baecher-Allan C., Kaskow B.J., Weiner H.L. (2018). Multiple Sclerosis: Mechanisms and Immunotherapy. Neuron.

[62] Nakahashi-oda C., Gamage K., Udayanga S., Nakamura Y., Nakazawa Y., Totsuka N., Miki H., Iino S., Tahara-hanaoka S., Honda S. (2016). Apoptotic epithelial cells control the abundance of T reg cells at barrier surfaces. Nat. Immunol..

[63] Kraus J., Ling A.K., Hamm S., Voigt K., Oschmann P., Engelhardt B. (2004). Interferon-beta stabilizes barrier characteristics of brain endothelial cells in vitro. Ann. Neurol..

[64] Boziki M.K., Kesidou E., Theotokis P., Mentis A.A., Karafoulidou E., Melnikov M. (2020). brain sciences Microbiome in Multiple Sclerosis: Where Are We, What We Know and Do Not Know. Brain Sci..

[65] Staun-Ram E., Miller A. (2010). Cathepsins (S and B) and their inhibitor Cystatin C in immune cells: Modulation by interferon-beta and role played in cell migration. J. Neuroimmunol..

[66] Barcutean L., Balasa R. (2024). Short and Medium Chain Fatty Acids in a Cohort of Naïve Multiple Sclerosis Patients: Pre- and Post- Interferon Beta Treatment Assessment. Biol. Targets Ther..

[67] Hidalgo-cantabrana C., Delgado S., Ruiz L., Ruas-madiedo P., Sánchez B., Margolles A. (2017). *Bifidobacteria* and Their Health-Promoting Effects. Microbiol. Spectr..

[68] Gutierrez A., Pucket B., Engevik M.A. (2023). Bifidobacterium and the intestinal mucus layer. Microbiome Res. Rep..

[69] Ghimire S., Lehman P.C., Aguilar L.S., Shahi S.K., Hoang J. (2025). Specific microbial ratio in the gut microbiome is associated with multiple sclerosis. Proc. Natl. Acad. Sci. USA.

[70] Rolfes L., Pfeuffer S., Huntemann N., Schmidt M., Su C., Skuljec J., Aslan D., Hackert J., Kleinschnitz K., Hagenacker T. (2022). Immunological consequences of cladribine treatment in multiple sclerosis: A real-world study. Mult. Scler. Relat. Disord..

[71] Wiendl H., Schmierer K., Hodgkinson S., Derfuss T., Chan A., Sellebjerg F., Achiron A., Montalban X., Prat A., De Stefano N. (2023). Specific Patterns of Immune Cell Dynamics May Explain the Early Onset and Prolonged Efficacy of Cladribine Tablets: A MAGNIFY-MS Substudy. Neurol. Neuroimmunol. Neuroinflamm..

[72] Kleinschnitz C., Skripuletz T., Pfeuffer S., Pawlitzki M., Rieckmann P., Penner I.-K., Knaup J., Wagner T., Hübschen M., Hellwig K. (2025). Is therapy-free remission a realistic goal with cladribine tablets in multiple sclerosis? New insights into the mechanism of action and clinical implications of immune reconstitution with cladribine tablets in MS therapy. J. Neurol..

[73] Wiendl H., Barkhof F., Montalban X., Achiron A., Derfuss T., Chan A. (2025). Blood biomarker dynamics in people with relapsing multiple sclerosis treated with cladribine tablets: Results of the 2-year MAGNIFY-MS study. Front. Immunol..

[74] Ford R.K., Juillard P., Hawke S., Grau G.E., Marsh-wakefield F. (2022). Cladribine Reduces Trans-Endothelial Migration of Memory T Cells across an In Vitro Blood—Brain Barrier. J. Clin. Med..

[75] Bronzini M., Maglione A., Rosso R., Matta M., Masuzzo F., Rolla S., Clerico M. (2023). Feeding the gut microbiome: Impact on multiple sclerosis. Front. Immunol..

[76] Dziedzic A., Saluk J. (2022). Probiotics and Commensal Gut Microbiota as the Effective Alternative Therapy for Multiple Sclerosis Patients Treatment. Int. J. Mol. Sci..

[77] Wrzosek L., Miquel S., Noordine M.L., Bouet S., Chevalier-Curt M.J., Robert V., Philippe C., Bridonneau C., Cherbuy C., Robbe-Masselot C. (2013). Bacteroides thetaiotaomicron and Faecalibacterium prausnitzii influence the production of mucus glycans and the development of goblet cells in the colonic epithelium of a gnotobiotic model rodent. BMC Biol..

[78] Miyake S., Kim S.S.W.S., Suda W., Oshima K., Nakamura M., Matsuoka T., Chihara N., Tomita A., Sato W., Kim S.S.W.S. (2015). Dysbiosis in the gut microbiota of patients with multiple sclerosis, with a striking depletion of species belonging to clostridia XIVa and IV clusters. PLoS ONE.

[79] Zhu Y., Chen B., Zhang X., Akbar M.T., Wu T., Zhang Y., Zhi L., Shen Q. (2024). Exploration of the Muribaculaceae Family in the Gut Microbiota: Diversity, Metabolism, and Function. Nutrients.

[80] Ding Y.H., Qian L.Y., Pang J., Lin J.Y., Xu Q., Wang L.H., Huang D.S., Zou H. (2017). The regulation of immune cells by Lactobacilli: A potential therapeutic target for anti-atherosclerosis therapy. Oncotarget.

[81] Rastogi S., Singh A. (2022). Gut microbiome and human health: Exploring how the probiotic genus Lactobacillus modulate immune responses. Front. Pharmacol..

[82] Kwon H.-K., Kim G.-C., Kim Y., Hwang W., Jash A., Sahoo A., Kim J.-E., Nam J.H., Im S.-H. (2013). Amelioration of experimental autoimmune encephalomyelitis by probiotic mixture is mediated by a shift in T helper cell immune response. Clin. Immunol..

[83] Zangeneh Z., Rostamin M., Motamedi H., Alvandi A., Abiri R. (2025). The potential effectiveness of probiotics in reducing multiple sclerosis progression in preclinical and clinical studies: A worldwide systematic review and meta-analysis. PLoS ONE.

[84] Blais L.L., Montgomery T.L., Amiel E., Deming P.B., Krementsov D.N. (2021). Probiotic and commensal gut microbial therapies in multiple sclerosis and its animal models: A comprehensive review. Gut Microbes.

[85] Tankou S.K., Regev K., Healy B.C., Tjon E., Laghi L., Cox L.M., Kivisäkk P., Pierre I.V., Hrishikesh L., Gandhi R. (2018). A probiotic modulates the microbiome and immunity in multiple sclerosis. Ann. Neurol..

[86] Golan D., Halhal B., Glass-Marmor L., Staun-Ram E., Rozenberg O., Lavi I., Dishon S., Barak M., Ish-Shalom S., Miller A. (2013). Vitamin D supplementation for patients with multiple sclerosis treated with interferon-beta: A randomized controlled trial assessing the effect on flu-like symptoms and immunomodulatory properties. BMC Neurol..

[87] Buttari F., Zagaglia S., Marciano L., Albanese M., Landi D., Nicoletti C.G., Mercuri N.B., Silvestrini M., Provinciali L., Marfia G.A. (2017). TRPV1 polymorphisms and risk of interferon β-induced flu-like syndrome in patients with relapsing-remitting multiple sclerosis. J. Neuroimmunol..

[88] Pereira L.G., Rodrigues P., Viero F.T., Frare J.M., Ramanzini G., Trevisan G. (2022). Interferon-Beta Injection in Multiple Sclerosis Patients Related to the In- duction of Headache and Flu-Like Pain Symptoms: A Systematic Review and Meta-Analysis of Randomised Controlled Trials. Curr. Neuropharmacol..

[89] Hadgkiss E.J., Jelinek G.A., Weiland T.J., Pereira N.G., Marck C.H., van der Meer D.M. (2015). The association of diet with quality of life, disability, and relapse rate in an international sample of people with multiple sclerosis. Nutr. Neurosci..

[90] Wolter M., Grant E.T., Boudaud M., Steimle A., Pereira G.V., Martens E.C., Desai M.S. (2021). Leveraging diet to engineer the gut microbiome. Nat. Rev. Gastroenterol. Hepatol..

[91] Džidić Krivić A., Begagić E., Hadžić S., Bećirović A., Bećirović E., Hibić H., Tandir Lihić L., Kadić Vukas S., Bečulić H., Kasapović T. (2025). Unveiling the Important Role of Gut Microbiota and Diet in Multiple Sclerosis. Brain Sci..

[92] Filipi M.L., Beavin J., Brillante R.T., Costello K., Hartley G.C., Hartley K., Namey M., Leary S.O., Remington G. (2014). Nurses ’ Perspective on Approaches to Limit Flu-Like Symptoms During Interferon Therapy for Multiple Sclerosis. Int. J. MS Care.

[93] La Carpiae F., Wojczyk B.S., Annavajhalae M.K., Rebbaa A., Culp-HiII4 R., D’AlessandroGt A., Freedberg D.E., Uhlemann A.-C., Hode E.A. (2019). Transfusional iron overload and intravenous iron infusions modify the mouse gut microbiota similarly to dietary iron. Biofilms Microbiomes.

[94] Nemati M.H., Yazdanpanah E., Kazemi R., Orooji N., Dadfar S., Oksenych V., Haghmorad D. (2025). Microbiota-Driven Mechanisms in Multiple Sclerosis: Pathogenesis, Therapeutic Strategies, and Biomarker Potential. Biology.

[95] Boussamet L., Rajoka M.S.R., Berthelot L. (2022). Microbiota, IgA and Multiple Sclerosis. Microorganisms.

[96] Pröbstel A., Zhou X., Baumann R., Wischnewski S., Rojas O.L., Sellrie K., Bischof A., Kim K., Ramesh A., Dandekar R. (2020). Gut microbiota-specific IgA+ B cells traffic to the CNS in active multiple sclerosis. Sci. Immunol..

[97] Rojas O.L., Pröbstel A.-K., Porfilio E.A., Wang A.A., Charabati M., Sun T., Lee D.S.W., Galicia G., Ramaglia V., Ward L.A. (2019). Recirculating Intestinal IgA-Producing Cells Regulate Neuroinflammation via IL-10. Cell.

[98] Sterlin D., Larsen M., Fadlallah J., Parizot C., Vignes M., Autaa G., Dorgham K., Juste C., Lepage P., Aboab J. (2021). Perturbed microbiota/immune homeostasis in multiple sclerosis. Neurol. Neuroimmunol. Neuroinflamm..

[99] Takeuchi T., Ohno H. (2022). IgA in human health and diseases: Potential regulator of commensal microbiota. Front. Immunol..

[100] Lycett M.J., Lea R.A., Maltby V.E., Min M., Lechner-Scott J. (2023). The effect of cladribine on immunoglobulin levels compared to B cell targeting therapies in multiple sclerosis. Mult. Scler. J. Exp. Transl. Clin..

[101] Thompson A.J., Banwell B.L., Barkhof F., Carroll W.M., Coetzee T., Comi G., Correale J., Fazekas F., Filippi M., Freedman M.S. (2018). Diagnosis of multiple sclerosis: 2017 revisions of the McDonald criteria. Lancet Neurol..

[102] Abu-Saad K., Endevelt R., Goldsmith R., Shimony T., Nitsan L., Shahar D.R., Keinan-Boker L., Ziv A., Kalter-Leibovici O. (2019). Adaptation and predictive utility of a Mediterranean diet screener score. Clin. Nutr..

[103] Schröder H., Fitó M., Estruch R., Martínez-González M.A., Corella D., Salas-Salvadó J., Lamuela-Raventós R., Ros E., Salaverría I., Fiol M. (2011). A short screener is valid for assessing Mediterranean diet adherence among older Spanish men and women. J. Nutr..

[104] Martínez-González M.A., García-Arellano A., Toledo E., Salas-Salvadó J., Buil-Cosiales P., Corella D., Covas M.I., Schröder H., Arós F., Gómez-Gracia E. (2012). A 14-item mediterranean diet assessment tool and obesity indexes among high-risk subjects: The PREDIMED trial. PLoS ONE.

[105] Shahar D., Shai I., Vardi H., Brener-Azrad A., Fraser D. (2003). Development of a semi-quantitative Food Frequency Questionnaire (FFQ) to assess dietary intake of multiethnic populations. Eur. J. Epidemiol..

[106] Shai I., Rosner B.A., Shahar D.R., Vardi H., Azrad A.B., Kanfi A., Schwarzfuchs D., Fraser D. (2005). DEARR study Dietary evaluation and attenuation of relative risk: Multiple comparisons between blood and urinary biomarkers, food frequency, and 24-h recall questionnaires: The DEARR study. J. Nutr..

[107] Shai I., Vardi H., Shahar D.R., Azrad A.B., Fraser D. (2003). Adaptation of international nutrition databases and data-entry system tools to a specific population. Public Health Nutr..

[108] Dhariwal A., Chong J., Habib S., King I.L., Agellon L.B., Xia J. (2017). MicrobiomeAnalyst: A web-based tool for comprehensive statistical, visual and meta-analysis of microbiome data. Nucleic Acids Res..

[109] Lu Y., Zhou G., Ewald J., Xia J. (2023). MicrobiomeAnalyst 2.0: Comprehensive statistical, functional and integrative analysis of microbiome data. Nucleic Acids Res..

[110] Nearing J.T., Douglas G.M., Hayes M.G., MacDonald J., Desai D.K., Allward N., Jones C.M.A., Wright R.J., Dhanani A.S., Comeau A.M. (2022). Microbiome differential abundance methods produce different results across 38 datasets. Nat. Commun..

