# Supplementary material for: Immunotherapy-Mediated Modulation of the Gut Microbiota in Multiple Sclerosis: The Effects of High-Efficacy (Cladribine) and Moderate-Efficacy (Interferon Beta-1a) Treatments
Source: Int J Mol Sci. 2026 Apr 14;27(8):3500. doi: 10.3390/ijms27083500 (PMC13116577; doi:10.3390/ijms27083500)
Supplement: Supplementary file 1 [file ijms-27-03500-s001.zip › Tables S4-7.pdf]

Table S4 Microbes exhibiting differential abundance between patients who develop IAE following INFβ-1a treatment versus patients who continue treatment throughout a 1-year follow-up

Microbes	FDR	FC/LDA	Highest in
**Species**			
Unc. Lactobacillaceae bacterium	[2]0.0001<br>[3]0.002	31.1	IAE
Ruminococcus gnavus CC55 001C	[2]0.0001<br>[1]0.049	30.6<br>40.4	IAE
Coprococcus sp HPP0074	[2]0.0009<br>[3]0.083	15.2	IAE
Parabacteroides johnsonii CL02T12C29	[2]0.0036<br>[4]0.0297	6.49<br>`LDA -2.34	IAE
Bifidobacterium sp MC_10	[2]0.018<br>[3]0.037<br>[4]0.019	4.22<br><br>LDA -2.07	IAE
Bifidobacterium animalis	[2]0.066<br>[3]0.083	2.94	IAE
Bacteroides thetaiotaomicron	[2]0.075<br>[3]0.077	0.07	No IAE
Bacteroides massiliensis B84634_=_Timone 84634 =DSM 17679= JCM13223	[2]0.093<br>[3]0.077	0.10	No IAE
Unc. Sarcina sp	[3]0.077<br>[4]0.026	 LDA -2.03	IAE
**Genus**			
Streptococcus	[2]0.00008<br>[1]0.0003	21.1<br>23.6	IAE
Ruminococcus gnavus group	[2]0.00008<br>[1]3x10^{-6}	35.6<br>116.0	IAE
Lactobacillus	[2]0.005<br>[3]0.063	13.5	IAE
Negativibacillus	[2]0.079<br>[3]0.005	4.0	IAE
Megamonas	[2]0.0098<br>[1]0.0002<br>[3]0.027	0.001<br>0.0002	No IAE
Prevotella 7	[2]`0.061<br>[3]0.005	0.01	No IAE
Alloprevotella	[2]0.065<br>[1]8x10^{-8}<br>[3]0.0025	0.01<br>1x10^{-7}	No IAE
Tyzzerella 4	[2]0.068<br>[1]0.067<br>[3]0.063	6.6<br>3.3	IAE
Fusicatenibacter	[1]0.008<br>[3]0.027	0.38	No IAE
Lachnospiraceae UCG_001	[1]0.028<br>[3]0.051<br>[4]0.032	0.27<br><br>LDA 3.3	No IAE
Sarcina	[1]0.081<br>[4]0.001	68.6<br>LDA -2.0	IAE
GCA 900066755	[3]0.042<br>[4]0.04	 LDA 1.46	No IAE
**Family**			
Streptococcaceae	[2]0.00002<br>[1]0.003	23.1<br>18.2	IAE

Lactobacillaceae	[2]0.009<br>0.054[3]	6.54	IAE
Puniceicoccaceae	0.012[2]<br>0.022[3]	5.17	IAE
Flavobacteriaceae	0.059[3]<br>0.027[4]	 LDA -1.14	IAE
**Order**			
Lactobacillales	0.00001[2]<br>0.003[1]	14.0<br>9.75	IAE
Opitutales	0.077[2]<br>0.005[3]	3.72	IAE
**Class**			
Bacilli	$1x10^{-8}$ [2]<br>0.001[1]	24.5<br>10.2	IAE

Microbes exhibiting differential relative abundance at baseline between patients who developed IAE leading to discontinuation of INFβ-1a therapy (n=5) versus patients who continue treatment throughout the 1-year follow-up (n=26). Significant by statistical method: [1] DeSeq2, [2] EdgeR, [3] MetagenomeSeq at FDA<0.1 or [4]LEfSe at *p*<0.05. Only microbes significantly differential as determined by at least two statistical methods are presented. Abbreviations: FC – fold change, FDR – false discovery rate, IAE – intolerable adverse events leading to drug discontinuation, LDA – linear discriminant analysis, LEfSe – LDA effect size, unc.- uncultured.

Table S5
Associations between Mediterranean diet adherence and clinical response in INFβ-1a- or CladT-treated patients

MDS<br>Mean ± SE	NEDA	DA	*p*-value
INFβ-1a patients<br>(1 year)	5.46±0.5	7.0±1.1	0.4
CladT patients<br>(1 year)	7.69±0.5	7.25±0.4	0.6
CladTpatients<br>(2 years)	6.86±0.5	7.5±0.4	0.6

Comparison of MDS at baseline in patients with NEDA vs. patients with DA, following 1 year INFβ-1a therapy or 1 and 2 years following CladT initiation (Mann-Whitney U test). Abbreviations: DA – disease activity, MDS – Mediterranean diet score - NEDA – no evidence of disease activity, SE – standard of error.

Table S6 Associations between nutrient intake and clinical response in INFβ-1a- or CladT- treated patients

	INFβ-1a patients (1 year)			CladT patients (1 year)			CladT patients (2 years)		
nutrient Mean ± SE	NEDA	DA	*p*-value	NEDA	DA	*p*-value	NEDA	DA	*p*-value
Fructose (gr)	17.6±3.7	25.9±3.5	0.038						
Trans fatty acids (gr)				0.14±0.05	0.27±0.08	0.034			
Calories carbohydrates (%)				45.0±1.8	39.4±1.7	0.044			
Calories fat (%)				36.8±1.3	40.5±1.1	0.019			
Calories saturated fat (%)				10.5±1.0	12.9±0.7	0.034			
Alcohol (gr)							2.99±1.2	0.49±0.2	0.016

Comparison of mean daily nutrient intake at baseline in patients maintaining NEDA versus patients developing disease activity, following 1 year of INFβ-1a therapy or 1 and 2 years of CladT therapy. Only significant results are presented (Mann-Whitney U test). Abbreviations: DA – disease activity, gr- gram, NEDA – no evidence of disease activity, SE – standard of error.

Table S7 Associations between nutrient intake at baseline and development of IAE in INFβ-1a-treated patients

Nutrient<br>Mean ± SE	IAE	Without IAE	*p*-value
Sodium (mg)	2771±500	4374±481	**0.035**
Protein (gr)	79.2±13.5	112±10.8	0.083
Carbohydrates (gr)	177±26.4	263±34.9	0.063
Food energy (Kcal)	1735±256	2452±272	0.071
Iron (mg)	9.56±1.4	14.0±1.4	**0.038**
Zinc (mg)	9.21±1.3	12.8±1.3	0.072
Copper (mg)	1.53±0.23	2.12±0.21	0.073
Vitamin E (mg)	9.80±1.52	13.7±1.48	0.088
Linolenic (gr)	2.15±0.40	3.13±0.39	0.099
Mono saturated fat (gr)	28.9±4.2	39.6±4.1	0.088
Poly saturated fat (gr)	16.3±3.1	23.3±2.5	0.097
Phenylalanine (gr)	3.38±0.58	4.79±0.46	0.082
Tryptophan (gr)	0.79±0.13	1.11±0.10	0.081
Arginine (gr)	4.03±0.67	5.61±0.50	0.085
Cysteine (gr)	0.89±0.15	1.32±0.14	0.054
Selenium (ug)	100±17.9	149±18.1	0.072
Added sugar (gr)	24.2±3.9	44.0±7.9	**0.034**

Comparison of mean daily nutrient intake at baseline in patients who developed intolerable adverse events (IAE) following INFβ-1a initiation, leading to discontinuation of therapy vs. patients who continued INFβ-1a treatment throughout follow-up. Only significant (**bold**) or near significant data presented (T-Test). Abbreviations*:* SE – standard of error.
